# Potential Bioactive Compounds from Seaweed for Diabetes Management

**DOI:** 10.3390/md13085447

**Published:** 2015-08-21

**Authors:** Yusrizam Sharifuddin, Yao-Xian Chin, Phaik-Eem Lim, Siew-Moi Phang

**Affiliations:** 1 Institute of Ocean and Earth Sciences, University of Malaya, 50603 Kuala Lumpur, Malaysia; E-Mails: chinyx1@gmail.com (Y.-X.C.); phaikeem@um.edu.my (P.-E.L.); phang@um.edu.my (S.-M.P.); 2 Institute of Biological Sciences, Faculty of Science, University of Malaya, 50603 Kuala Lumpur, Malaysia

**Keywords:** α-glucosidase, aldose reductase, algae, antioxidant, biotechnology, diabetes, DPP-4, GIP, PTP1B, seaweed

## Abstract

Diabetes mellitus is a group of metabolic disorders of the endocrine system characterised by hyperglycaemia. Type II diabetes mellitus (T2DM) constitutes the majority of diabetes cases around the world and are due to unhealthy diet, sedentary lifestyle, as well as rise of obesity in the population, which warrants the search for new preventive and treatment strategies. Improved comprehension of T2DM pathophysiology provided various new agents and approaches against T2DM including via nutritional and lifestyle interventions. Seaweeds are rich in dietary fibres, unsaturated fatty acids, and polyphenolic compounds. Many of these seaweed compositions have been reported to be beneficial to human health including in managing diabetes. In this review, we discussed the diversity of seaweed composition and bioactive compounds which are potentially useful in preventing or managing T2DM by targeting various pharmacologically relevant routes including inhibition of enzymes such as α-glucosidase, α-amylase, lipase, aldose reductase, protein tyrosine phosphatase 1B (PTP1B) and dipeptidyl-peptidase-4 (DPP-4). Other mechanisms of action identified, such as anti-inflammatory, induction of hepatic antioxidant enzymes’ activities, stimulation of glucose transport and incretin hormones release, as well as β-cell cytoprotection, were also discussed by taking into consideration numerous *in vitro*, *in vivo*, and human studies involving seaweed and seaweed-derived agents.

## 1. Introduction

### 1.1. Definition and Current Incidence of Diabetes

Diabetes mellitus is a group of chronic diseases, which can be attributed to hyperglycaemia, a condition characterised by an excessive concentration of glucose circulating in the blood. Diabetes mellitus can be categorised into two main forms namely Type I diabetes mellitus (T1DM), caused by the absolute absence of insulin production due to auto-immune mediated disintegration of pancreatic β-cells, and Type II diabetes mellitus (T2DM), which is due to the relative deficiency of the same hormone involving insulin resistance, aberrant synthesis of hepatic glucose and progressive deterioration of pancreatic β-cell functions [1]. T2DM sufferers are not dependent on insulin injection, unlike those with T1DM, if diet and hypoglycaemic agents were sufficient for effective glycaemic control. The World Health Organisation (WHO) had projected the total number of people with diabetes mellitus (DM) worldwide to increase from 171 million in 2000 to nearly 370 million in 2030, with the prevalence of the disease for all age groups to be 4.4% in 2030, compared with 2.8% in 2000 [2]. Diabetes is frequently correlated with increased risk in hypertension, macrovascular and microvascular complications, blindness and kidney failure [3]. Macrovascular complications have been observed to be higher in T2DM patients with the risk of developing diseases involving the human vascular tree such as stroke, coronary artery disease and peripheral arterial disease to be fourfold higher, which developed relatively earlier in diabetic than non-diabetic patients [4,5]. Sufferers of T2DM normally have reduced life expectancy due to these various co-morbidities [6,7].

### 1.2. Pathophysiology and Prevalence of Type II Diabetes Mellitus (T2DM)

T2DM accounts for approximately 90% of diabetes cases worldwide, verging on epidemic proportions affecting both developed and developing countries [8]. This increase is attributed to greater prevalence of sedentary lifestyle, unhealthy diet and rise of obesity within modern society as well as an increasing number of elderly populations [2]. Furthermore, the pathophysiological processes leading to T2DM including deterioration of β-cells functions, chronic hyperglycaemia, and insulin resistance in musculoskeletal and adipose tissues [9,10] may be latently present for a considerably long period, prior to any diagnosis or manifestations of medical complications. Often, β-cell functions are reduced to approximately 50% by the time diabetes is diagnosed [11]. In normal individuals, a fairly constant level of insulin is released into the bloodstream by pancreatic β-cells, which will increase the blood level of the hormone upon food ingestion. Post-prandial insulin release, as well as increased blood glucose level, inhibit renal and hepatic secretion of glucagon into circulation, effecting glucose uptake into various tissues. Individuals with post-prandial hyperglycaemia exhibit decreased insulin secretion after food consumption and less inhibition on the release of glucagon, leading to aberrant hepatic and renal glucose production, reduced glucose uptake by cells and consequently elevated blood glucose levels [12,13]. Progressive entry of ingested and endogenous glucose into the circulation that outpace the removal rate causes prolonged high concentrations of blood glucose, leading to loss of homeostatic post-prandial glycaemic control followed by hyperglycaemia [12,13,14].

Preventing this costly lifestyle disease is more desirable than treating its various associated complications and in light of the rapid escalation of T2DM cases globally, urgent action in identifying new prevention strategies are needed. Prevention is crucial due to the premature morbidity and mortality associated with the disease that could potentially burden personal, as well as annual national, healthcare expenditures. Previous major studies have demonstrated the beneficial values of glycaemia control in preventing and/or reducing the risk of microvascular complications related to diabetes such as neuropathy, nephropathy and retinopathy as well as various macrovascular complications [15,16]. Other subsequent large-scale studies also showed the positive effect of lifestyle intervention approaches [17,18]. Indeed, the primary objective in T2DM management is to ensure sufficient delivery of glucose to various tissues of the body and also to delay, and/or prevent associated complications of T2DM due to hyperglycaemia by achieving good glycaemic control. Current medical care employs a wide array of pharmacological and lifestyle intervention approaches aimed at managing hyperglycaemia. Lifestyle interventions consisting of suitable diet and physical exercise decreased the incidence of diabetes by up to 58% [19,20] and long-term glycaemic control was found to be beneficial in reducing the risk of both micro- and macrovascular complications in T2DM patients [21].

Understanding the complexity of glucose homeostasis, diabetes pathophysiology and other associated risk factors such as obesity is important considering the multi-factorial nature of the disease. Nutrition has been regarded to play a pivotal role within this complex pathophysiology of T2DM and in the last several years, increasing amount of evidence has emerged linking several nutrients and food sources in positively managing T2DM. Previous studies have suggested that high dietary intake of fruit, whole-grain, and vegetables may confer protection against or reduced the risk of T2DM development [22,23].

### 1.3. Seaweed Consumption and Diabetes

Seaweed have been traditionally consumed as a readily available wholefood especially among coastal communities particularly in Asia [24,25], for example the Japanese were reported to consume seaweed in their daily diet approximately 5.3 g per day [26]. Additionally, seaweed has also been prescribed for numerous ailments in different Asian traditional medical systems. Although consumer awareness and dietary seaweed intake are generally low in other regions [27], popularisation of East-Asian diet worldwide has gradually increased public interest and acceptance of seaweed as a food source, partly due to their suggested health benefits. As adoption of good nutritional habits as part of a healthy lifestyle are currently in vogue and concomitant increase of consumers’ market influence on the food industry, consumption of seaweed and seaweed-based products are rising similar to the trend observed with fresh fruits and vegetables. In other parts of the world, seaweed use is generally limited to extracts and food additives [28], as well as isolates, such as carrageenan and alginate, which are normally used in various applications [29]. Currently, there is a growing awareness on the role of food beyond the basic nutritional value by providing health benefits and reducing the risk of various illnesses including diabetes [30,31]. High consumption of seaweed in daily diet has been associated with lower risk of diseases, such as cardiovascular disease and hyperlipidaemia [32], as well as breast cancer [33]. Furthermore dietary changes involving reduction of daily seaweed consumption due to a more Westernised diet in Asian societies that traditionally consumed seaweed, indicated increased incidence of chronic lifestyle diseases, hence highlighting seaweed’s health benefits [34,35,36]. Indeed, seaweed have often been overlooked as a good source of functional food and are under-utilised dietary source of novel as well as structurally diverse bioactive compounds with high biomedical potential that are not commonly present in terrestrial plants. In this review paper, we aim to narrate the diversity of beneficial contents and bioactive compounds from seaweed, which are potentially useful in preventing or managing T2DM via various pharmacologically relevant targets.

Seaweeds are rich in bioactive compounds in the form of polyphenols, carotenoids, vitamins, phycobilins, phycocyanins, and polysaccharides, among others, and many of these are known to possess beneficial applications in human health [27]. There is also an incomparably rich amount of minerals and trace elements content in seaweed due to their ability in retaining inorganic marine substances attributed to the features of their cell surface polysaccharides where several of these essential minerals can be found at relatively higher levels than in terrestrial food sources [37,38,39]. Some seaweed species may contain minerals in more than 30% of their dry weight [40] and all of the essential minerals and trace elements required for healthy human diet can be found in seaweed [37]. Edible seaweed are also low in calories and rich in dietary fibre, unsaturated fatty acids and vitamins [41,42], making them suitable for managing diabetes. Indeed, dietary consumption of *Porphyra yezoensis* and *Undaria pinnatifida* was associated with low incidence of diabetes in Korean men [43]. A nationally representative survey conducted on health and nutrition, suggests that the risk of developing T2DM in Korean men may be reduced by dietary consumption of seaweed [44]. The consumption of commercial blend of *Ascophyllum nodosum* and *Fucus vesiculosus* was associated with improved insulin regulation and sensitivity, measured in human subjects using the Cederholm index upon carbohydrate ingestion, compared with placebo [45]. Consumption of mekabu (sporophylls of *Undaria pinnatifida*) with a white rice-based breakfast by healthy volunteers demonstrated a reduction of post-prandial glucose concentration and this was attributed to the content and viscosity of fucoxanthin in mekabu [46]. Recently, a study involving more than 4000 participants in Korea revealed that insulin level and insulin resistance were inversely associated with dietary intake of flavonols and flavones, thereby reducing the risk of T2DM [47]. However, not all of these studies fully elucidate the factors involved in beneficial properties of seaweed dietary intake in managing diabetes. Therefore, the potential of various beneficial components in seaweed and their possible modes of action against the development of T2DM deserve a closer investigation.

## 2. Seaweed Composition and Effects on Diabetic Targets

### 2.1. Unsaturated Fatty Acids from Seaweed

Seaweeds are rich in unsaturated fatty acids. Fatty acids containing two or more methylene-interrupted double bonds are important for normal cellular functions and have gained worldwide interest in their utilisation as nutraceuticals including against T2DM.

#### 2.1.1. Monounsaturated Fatty Acids (MUFA) from Seaweed

The substitution of saturated fatty acids with monounsaturated fatty acids (MUFA) was found to improve insulin sensitivity in healthy and glucose-intolerant subjects [48] with no excessive total fat intake [49]. Even though the suggested positive effects of MUFA were not found by other earlier studies [50,51], they are generally considered as a useful source of fat for sufferers with varying degrees of insulin resistance [52]. The exact mechanisms of MUFA in diabetes management are still not fully elucidated but several possible modes of action have been suggested including promoting glucose uptake by up-regulating glucose transporter type 1 (GLUT1) and type 4 (GLUT4) in the cell membrane [53], as well as possessing cytoprotective effects on pancreatic β-cells. Furthermore, the beneficial effect of dietary MUFA in improving insulin sensitivity in rats was attributed to the preservation of IRS/PI3K insulin pathway and increased GLUT4 translocation to the cell membrane [54]. MUFA has also been linked to changes in incretin responses and gastric emptying, where dietary MUFA elevated the glucagon-like peptide (GLP-1) in both healthy and diabetic subjects [55]. In addition, the intake of dietary MUFA increased the levels of adiponectin [56], which is associated with reduced risk of T2DM [57]. Wang and colleagues reported that the extracts and MUFA derivatives isolated from the green seaweed *Ulva lactuca* induced many antioxidant-response element (ARE)-driven antioxidant genes in various mouse tissues [58]. Antioxidant, anti-inflammatory properties and stimulation of hepatic antioxidant enzymes have been reported as one of the anti-diabetic potential of seaweed as discussed in the later section of this review.

#### 2.1.2. Polyunsaturated Fatty Acids (PUFA) from Seaweed

Comparable to MUFA, polyunsaturated fatty acids (PUFA) also play various important biological functions both structural and physiological in nature [59]. They are also involved in cellular and tissue metabolism including thermal adaptation and membrane fluidity regulation [60]. Furthermore, increased public interest in healthy lifestyle and diet had propelled PUFA into popular market demands [61]. The potential of seaweed as a good source of PUFA has been well documented [62,63,64,65]. Seaweed may contain lipids, which represent up to 2% in algal dry weight and primarily PUFA [62,66]. Majority of the PUFA present in seaweed are in the form of omega-3 and omega-6 fatty acids, both are essential in human diet and generally present in an almost frequent ratio [62].

Various edible seaweeds, such as *Undaria pinnatifida*, *Himanthalia elongata*, and *Laminaria ochroleuca*, contain higher percentage of unsaturated fatty acids (MUFA and PUFA) compared with saturated fatty acids, with *U. pinnatifida* containing almost 70% of fatty acids as PUFA [62]. A balanced diet should consists of omega-3 and omega-6 fatty acids in a suitable ratio and ideal ratio should range from 1:3 to 1:5 of omega-3 to omega-6 fatty acids, as it affects the ratio of resultant eicosanoids [38,67]. Many seaweed species from the Phaeophyta and Rhodophyta phyla contain higher concentrations of unsaturated fatty acids compared with those from Chlorophyta, with the exception of *Ulva* sp., which possesses high concentrations of omega-3 fatty acids [65,68]. Furthermore according to Pereira and colleagues, almost all seaweed species they had studied encompassing the three phyla can be regarded as a good source of dietary PUFA as their omega-6 and omega-3 fatty acids ratio ranged from 0.29 to 6.73, which is within the recommended value [65]. Omega-6 PUFA from plant sources exhibit positive effects on insulin sensitivity [69] and are linked to lower risk of developing T2DM [70,71,72]. Diet rich in omega-6 PUFA was shown to improve insulin sensitivity in human subjects during a five-week study [69]. Just as observed with MUFA, PUFA also affects insulin action by changing physical properties of cellular membranes such as elevating binding affinity of the insulin receptor and increasing glucose uptake by cells via glucose transporters [73,74,75]. Omega-3 PUFA may also reduce insulin resistance via other mechanisms including decreasing circulating triglycerides and low-density lipoprotein particles [76] as well as being anti-inflammatory via toll-like receptors (TLR) inhibition. TLR-2 and TLR-4 can be inhibited by omega-3 PUFA [77] and long-chain omega-3 PUFA have been associated with increased levels of anti-inflammatory cytokines including interleukin-6r and interleukin-10 (IL-10), as well as lower concentration of pro-inflammatory interleukin-1ra and tumour necrosis factor-α (TNF-α) [78]. Omega-3 PUFA supplementation reduced triglycerides concentration, blood pressure and inflammatory markers in T2DM patients [79]. Khan and colleagues screened 37 species of seaweed representative of three different phyla collected from Korean coast for their anti-inflammatory activities measured by potential inhibition of mouse ear erythema and edema [80]. The methanolic extracts of an edible species of brown seaweed popular in Korean traditional medicine, *Undaria pinnatifida* and a species of green seaweed, *Ulva linza* exhibited the best inhibitory activities against inflammatory response attributable to their PUFA content [80]. Furthermore, omega-3 PUFA also influenced expression of several genes that are involved in lipid and carbohydrate metabolism by affecting their expression or the activity of several transcription factors including hepatic nuclear factors, sterol-regulatory element-binding protein-1c and liver X receptor [81].

### 2.2. Dietary Fibres from Seaweed

Consumption of dietary fibre has been associated with weight loss due to prolonged gastric clearance rate leading to increased satiety and concomitant reduction of food intake. Previous large-scale prospective observation studies have demonstrated that high intakes of dietary fibre are consistently correlated to a markedly reduced incidence of T2DM [82,83] and many international organisations issued guidelines recommending high daily intakes of dietary fibres, ranging from at least 30 g/day for healthy individuals to 50 g/day for diabetic patients [84,85]. Many seaweed species contain similar or higher total fibre content compared with their terrestrial counterparts [38]. For example, *Himanthalia elongata*, *Ascophyllum nodosum*, *Laminaria digitata* and *Palmaria palmata* contain higher percentage of total dietary fibre and lower soluble carbohydrate (g/100 g weight) compared with brown rice and bananas [38]. Furthermore, 8 g seaweed per serving can potentially provide close to 12.5% of an individual’s daily fibre requirement suggesting high-fibre intake with low glycaemic load is achievable with small quantity of seaweed consumption, hence its suitability for T2DM management [38]. The total dietary fibre intake by diabetic patients given seaweed supplements containing *Undaria pinnatifida* and *Saccharina japonica* for four weeks was observed to be 2.5 times higher compared with control, accompanied by concomitant improvements in other parameters including reduced glucose and blood lipid levels, as well as increased antioxidant enzyme activities [86].

Many observation studies, if not all, demonstrated an inverse relationship between dietary fibre intake and body weight [87,88] where various possible modes of action have been proposed as reviewed previously [89], including improving satiety, interactions with gastrointestinal hormones, depressing post-prandial lipaemia, reduced glycaemic and insulin levels, as well as attenuation of low grade inflammation [82,83,89,90,91,92]. Furthermore, increased total dietary fibre consumption reduced insulin resistance biomarkers [93]. Human subjects consuming arroz-caldo (porridge) incorporated with lambda-carrageenan showed significantly lower post-prandial glycaemic responses compared with control, suggesting the beneficial effect of carrageenan and dietary fibre in managing metabolic diseases such as diabetes [94]. The consumption of Nori (*Porphyra*) was demonstrated to reduce sharp glucose peak and decreased glycaemic response upon consumption of carbohydrate (white bread) by healthy volunteers from 100% to 68% [95]. Crude polysaccharides obtained from *Himanthalia elongata* significantly reduced blood glucose levels in alloxan-induced diabetic rabbits by approximately 50% after 8 h of 5 mg/kg intravenous administration [96] and further study revealed that fucan from *Himanthalia elongata* reduced blood glucose levels in diabetic rabbits [97]. Vaugelade and colleagues examined the effect of different seaweed-derived dietary fibres of varied viscosities extracted from three different species namely *Laminaria digitata*, *Palmaria palmata*, and *Eucheuma cottonii*, on intestinal absorption of glucose and insulin response in pigs [98]. Among the various non-starch polysaccharides evaluated, only the alginates from *L. digitata* by virtue of its relatively high viscosity compared with carrageenans from *E. cottonii* and xylans from *P. palmata*, significantly reduced blood glucose and insulin responses leading to 50% reduction of glucose absorption balance in pigs [98]. Polysaccharides derived from seaweed were also reported to be effective as stimulant of insulin secretion particularly low molecular weight oligosaccharides approximately 3 kDa in size [99].

Several studies also suggested increased post-prandial satiety in human subjects who consumed high dietary fibres [88] but these observations were not reproduced by other later studies [100,101,102]. The secretion of various gastrointestinal hormones including ghrelin and cholecystokinin which may play a role in achieving satiety, could possibly be influenced by dietary fibre intake [103,104,105,106]. High-fibre diet may lead to moderate reduction in body weight [88] and contribute to reduce diabetes risk especially with soluble dietary fibre [89], possibly by slowing of gastric emptying rate and reducing post-prandial glucose responses. Indeed, viscosity is important in the regulation of appetite and food intake by dietary fibres [107]. Several studies showed contradictory results on the effect of alginate on induction of satiety feeling and energy intake in human subjects but these discrepancies between the different studies can be attributed to the experimental design [108,109,110,111]. Pre-prandial intake of sodium alginate by normal, over-weight and obese individuals showed reduced mean daily intake of sugar, carbohydrate, protein, fat and saturated fat as well as decreased mean daily energy intake by 7%, indicating a potentially beneficial role of the gelling properties of sodium alginate formulation in managing obesity and T2DM [111]. The strong-gelling property of alginate consumed as a drink by volunteers selectively restored the uptake of glucose and cholesterol in overweight and obese individuals to the levels normally observed in healthy subjects [112]. Other previous reports on alginate-based supplements showed significant reduction of blood glucose levels [113,114]. Furthermore, colonic fermentation of alginate generated short chain fatty acids including propionate [115] which can modify cholesterol metabolism, decreased glucose absorption and reduced body weight [111,112,116]. In contrast, the effect of alginate intake for a 10-day period was found to have no effects on satiety, appetite, gastric motor functions and several gut satiety hormones in overweight and obese subjects [117]. Even though the results obtained were rather contradictory, Georg Jensen and colleagues suggested that consumption of high-dose alginate pre-load improved satiety feelings and reduced energy intake suggesting its beneficial role in facilitating weight loss, with further studies into the suitable volume of alginate needed for its suitability in short-term energy regulation [118]. Considering the possible link between abnormal glucose levels and the accumulation of advanced glycation end-products (AGE) in blood and tissues, alginate was also shown to inhibit the fructation of human serum albumin [119]. AGE has been implicated in diabetic microvascular complications.

Significant reduction in short-term energy intake following consumption of bread enriched with whole brown seaweed, *Ascophyllum nodosum* compared with control was reported but no effects on cholesterol, glucose, satiety or hunger were observed [120]. The absence of satiety effect associated with gelation and uptake of glucose and cholesterol in this study was attributed to the low amount of alginate present in the *A. nodosum*-enriched bread, whereas the reduced energy intake was due to gastric stretching effects [120].

High consumption of dietary fibres can also contribute to attenuation of inflammatory biomarkers although the exact mechanism(s) of action is yet to be fully elucidated [121]. Various possible mechanisms have been previously suggested including effects on lipid oxidation, anti-hyperglycaemic properties, presence of butyrate due to dietary fibre fermentation, reduced body weight [121] and direct immunomodulatory effect via specific binding to immune cell receptors [122]. The protective effects of high fibre diet on attenuation of inflammation may be partly explained by the reduction of oxidative stress caused by hyperglycemia, dyslipidemia as well as improvement of insulin sensitivity [123]. Mechanistic study on the role of fucoidan, a sulphated polysaccharide extracted from *Undaria pinnatifida*, as a potential therapeutic agent against obesity and diabetes via anti-inflammatory activities showed that fucoidan was able to repress the differentiation of adipose cells by inhibiting key adipocyte biomarkers, inflammatory cytokines and overproduction of reactive oxygen species (ROS) [124]. Inflammation and subsequent dysfunction of adipose tissue involves a combination of intrinsic and extrinsic cellular stress factors [125] subsequently contributing to development of insulin resistance in obese individuals.

The potential of *Ulva fasciata* as suitable alternative source for dietary fibre and effectively controlling the cholesterol level as part of low-calorie diet in managing body weight was demonstrated by [28] and an almost similar study on the nutritional benefits of *Ulva rigida* was reported by [126]. Another study involving the seaweed genus *Ulva* by BelHadj and colleagues, assessing the effect of polysaccharides extracted from *Ulva lactuca* on important enzymes related to obesity and diabetes [127]. The study found that the polysaccharides inhibited crucial enzymes involved in the metabolism of carbohydrate and fat, both in blood and small intestine [127]. Furthermore, the *Ulva*-derived polysaccharides also protected liver-kidney functions as observed by reduced activities of several liver health biomarkers including alanine transaminase (ALT) and aspartate transaminase (AST) [127]. Although the exact mechanistic data are still lacking, the mechanisms involved in the reported protective properties of high dietary fibre intake are either due to soluble viscous dietary fibre consumption or are shared by both soluble and insoluble dietary fibres. Furthermore, it is important to consider that although many previous studies attributed the beneficial effects observed to dietary fibres, these studies used algal extracts or whole seaweed which contain other seaweed compositions including protein, vitamins and minerals among others, which may contribute to the reported effects either directly or indirectly. More studies involving isolated and purified polysaccharides from seaweed are required in the future.

Therefore, seaweed composition primarily MUFA, PUFA and dietary fibres have been intensely studied for their anti-diabetic properties. There are relatively more studies involving the beneficial role of seaweed dietary fibres compared with those on unsaturated fatty acids, which remain to be fully explored. Seaweed dietary fibres were shown to improve post-prandial satiety feeling, decreased short-term energy intake, reduction of blood glucose levels and improved insulin sensitivity. Seaweed dietary fibres are also helpful in reducing body weight or weight maintenance, hence are beneficial in attenuating the risk of obesity. As obesity is linked to chronic, low inflammatory condition leading to insulin resistance and β-cell damage, preventing obesity and the associated inflammatory environment is desirable. Dietary fibres, MUFA, and PUFA from different seaweed species have been reported to be beneficial in mitigating inflammatory response due to hyperglycaemia and adiposity.

## 3. Diverse Anti-Diabetic Properties of Seaweed

Research on anti-diabetic potential of seaweed is generally non homogeneous as certain seaweed components, bioactive compounds and mechanisms of action have been studied relatively more extensive than others. Apart from unsaturated fatty acids and dietary fibres, studies on anti-diabetic properties involving polyphenols from seaweed are considerably more prominent with a variety of polyphenolic compounds isolated against many well-known anti-diabetic targets. Polyphenolic compounds are known to form complexes upon interaction with numerous proteins [128] and those derived from vegetables and fruits exhibit various activities including anti-diabetes [129]. In this section of the review, we attempt to discuss the anti-diabetic targets of seaweed bioactive compounds including inhibition of enzymes involved in maintenance of glucose homeostasis such as α-amylase, α-glucosidase, aldose reductase and protein tyrosine phosphatase 1B (PTP1B), inhibition of incretin hormones activities, promotion of glucose uptake by cells, anti-obesity, as well as anti-inflammation and cytoprotection of β-cells.

### 3.1. Reduction of Glucose Levels via Inhibition of α-Glucosidase and α-Amylase Activities

Starch digestion in mammals is primarily via α-amylase and α-glucosidase in the small intestine and abrupt post-prandial elevation of blood glucose concentrations is due to hydrolysis of carbohydrates. Maltose and isomaltose produced by α-amylase action are subsequently hydrolysed by α-glucosidase, which is a membrane-bound enzyme present at the epithelium of the small intestine, yielding glucose [130]. Inhibition of both α-amylase and α-glucosidase activities can profoundly attenuate post-prandial increase of blood glucose following a mixed carbohydrate intake and is a logical approach in managing glucose level for borderline and T2DM sufferers. Numerous extracts and bioactive compounds isolated from seaweed were found to significantly inhibit the activities of these carbohydrate hydrolytic enzymes.

Table 1 lists bioactive compounds from various seaweed species, which demonstrated inhibition of α-amylase and α-glucosidase activities. Acetone crude extract of *Caulerpa racemosa* and *Spatoglossum schroederi* inhibited the α-amylase activity *in vitro* with ED_50_ of 0.09 mg/mL and 0.58 mg/mL, respectively [131]. Crude water extract of *Halimeda macroloba* inhibited α-glucosidase activity *in vitro* with IC_50_ value of 6.388 mg/mL [132]. Crude water extracts of several brown seaweed species including *Padina sulcata*, *Sargassum binderi* and *Turbinaria conoides* were also demonstrated to inhibit α-glucosidase activity [132]. Good α-amylase inhibitory activity by several phloroglucinol derivatives from *Eisenia bicyclis* at 1 mM were reported with 97.5% and 87.5% inhibition by dieckol and eckol respectively [133]. Inhibitory potential of fucofuroeckol A from *E. bicyclis* is more potent against α-amylase (IC_50_ = 42.91 μM) compared with dioxinodehydroeckol (IC_50_ = 472.70 μM), but is weaker against α-glucosidase compared with the latter [134]. Several bromophenols isolated from red seaweed *Odonthalia corymbifera* showed strong inhibition against *Saccharomyces cerevisiae* α-glucosidase activity with IC_50_ values ranging from 0.098 μM to 89.0 μM, with bis(2,3-dibromo-4,5-dihydroxybenzyl) ether (BDDE) found to be the most potent [135]. Bromophenols from another red seaweed species *Symphyocladia latiuscula* also strongly inhibited α-glucosidase of *S. cerevisiae*, with the most profound inhibition shown by BDDE (IC_50_ = 0.03 μM) followed by 2,3,6-tribromo-4,5-dihydroxybenzyl alcohol (IC_50_ = 11.0 μM) [135]. The two bromophenols from *S. latiuscula* also inhibited the activities of rat intestinal maltase and sucrase but with lower inhibitory potencies as shown in Table 1.

**Table 1:** Glucose levels reduction via inhibition of α-amylase and α-glucosidase activities.

Seaweed	Active Agent(s)	Activity	Test System(s)	References
*C. racemosa* *S. schroederi*	Acetone crude extract	α-amylase inhibition, ED_50_ = 0.09 mg/mL α-amylase inhibition, ED_50_ = 0.58 mg/mL	*In vitro* assay	[131]
*E. stolonifera*	Water extract	α-glucosidase inhibition against: α-glucosidase (*Saccharomyces*), IC_50_ = 0.026 mg/mL Rat intestinal maltase, IC_50_ = 4.213 mg/mL Rat instestinal sucrase, IC_50_ = 10.10 mg/mL Rat intestinal isomaltase, IC_50_ ˃ 100 mg/mL Rat intestinal glucoamylase, IC_50_ ˃ 100 mg/mL	*In vitro* assay	[136]
*E. stolonifera*	Methanolic extract	α-glucosidase inhibition against: α-glucosidase (*Saccharomyces*), IC_50_ = 0.022 mg/mL Rat intestinal maltase, IC_50_ = 0.772 mg/mL Rat instestinal sucrase, IC_50_ = 4.056 mg/mL Rat intestinal isomaltase, IC_50_ ˃ 100 mg/mL Rat intestinal glucoamylase, IC_50_ = 5.851 mg/mL	*In vitro* assay	[136]
*O. corymbifera*	Bromophenols	α-glucosidase (*S. cerevisiae*) inhibition by: BDDE, IC_50_ = 0.098 μM 4-Bromo-2,3-dihydroxy-6-hydroxymethylphenyl 2,5- dibromo-6-hydroxy-3-hydroxymethylphenyl ether, IC_50_ = 25.0 μM 4-Bromo-2,3-dihydroxy-6-methoxymethylphenyl 2,5-dibromo-6-hydroxy-3-methoxymethylphenyl ether, IC_50_ = 53.0 μM 2,3-dibromo-4,5-dihydroxybenzyl alcohol, IC_50_ = 89.0 μM	*In vitro* assay	[135]
*P. lancifolia*	BDDE	α-glucosidases inhibition against: *B. stearothermophilus*, IC_50_ = 0.12 μM *S. cerevisiae*, IC_50_ = 0.098 μM Rat intestinal maltase, IC_50_ = 1.20 mM Rat instestinal sucrase, IC_50_ = 1.00 mM	*In vitro* assay	[137]
*S. latiuscula*	Bromophenols	α-glucosidase (yeast) inhibition by: BDDE, IC_50_ = 0.03 μM 2,3,6-Tribromo-4,5-dihydroxybenzyl Alcohol, IC_50_ = 11.0 μM	*In vitro* assay	[135]
*S. latiuscula*	Bromophenols	BDDE: Sucrase inhibition, IC_50_ = 2.4 mM Maltase inhibition, IC_50_ = 3.2 mM 2,3,6-Tribromo-4,5-dihydroxybenzyl Alcohol: Sucrase inhibition, IC_50_ = 4.2 mM Maltase inhibition, IC_50_ ˃ 5.0 mM	*In vitro* assay	[135]
*E. bicyclis*	Phloroglucinol derivatives	α-amylase inhibition at 1 mM by: Dieckol, inhibition = 97.5% 1-(3′,5′-dihydroxyphenoxy)-7-(2″,4″,6″-trihydroxyphenoxy)-2,4,9-trihydroxydibenzo-1,4-Dioxin, inhibition = 89.5% Eckol, inhibition = 87.5%	*In vitro* assay	[133]
*E. stolonifera* *E. bicyclis*	Phlorofucofuroeckol-A Dieckol 7-Phloroeckol Eckol Dioxinodehydroeckol Phloroglucinol	α-glucosidase inhibition by: Phlorofucofuroeckol-A, IC_50_ = 1.37 μM Dieckol, IC_50_ = 1.61 μM 7-Phloroeckol, IC_50_ = 6.13 μM Eckol, IC_50_ = 22.78 μM Dioxinodehydroeckol, IC_50_ = 34.60 μM Phloroglucinol, IC_50_ = 141.18 μM	*In vitro* assay	[138]
*E. bicyclis*	Fucofuroeckol A	α-amylase inhibition, IC_50_ = 42.91 μM α-glucosidase inhibition, IC_50_ = 131.34 nM	*In vitro* assay	[134]
*E. bicyclis*	Dioxinodehydroeckol	α-amylase inhibition, IC_50_ = 472.70 μM α-glucosidase inhibition, IC_50_ = 93.33 nM	*In vitro* assay	[134]
*E. cava*	Dieckol Fucodiphloroethol G Phlorofucofuroeckol A 6,6′-bieckol 7-Phloroeckol	α-glucosidase inhibition by: Dieckol, IC_50_ = 10.8 μM Fucodiphloroethol G, IC_50_ = 19.5 μM Phlorofucofuroeckol A, IC_50_ = 19.7 μM 6,6′-bieckol, IC_50_ = 22.2 μM 7-Phloroeckol, IC_50_ = 49.5 μM	*In vitro* assay	[139]
*E. cava*	Dieckol 7-Phloroeckol 6,6′-bieckol Fucodiphloroethol G Phlorofucofuroeckol A	α-amylase inhibition by: Dieckol, IC_50_ = 124.9 μM 7-Phloroeckol, IC_50_ = 250.0 μM 6,6′-bieckol, IC_50_ ˃ 500 μM Fucodiphloroethol G, IC_50_ ˃ 500 μM Phlorofucofuroeckol A, IC_50_ ˃ 500 μM	*In vitro* assay	[139]
*E. cava*	Dieckol	α-amylase inhibition, IC_50_ = 0.66 mM α-glucosidase inhibition, IC_50_ = 0.24 mM	*In vitro* assay	[140]
*E. maxima*	Eckol Dibenzo [1,4] dioxine-2,4,7,9-tetraol Phloroglucinol	α-glucosidase inhibition by: Eckol, IC_50_ = 11.16 μM Dibenzo[1,4]dioxine-2,4,7,9-tetraol, IC_50_ = 33.69 μM Phloroglucinol, IC_50_ = 1991 μM	*In vitro* assay	[141]
*G. elliptica*	2,4,6-tribromophenol	α-glucosidase inhibition against: *B. stearothermophilus*, IC_50_ = 130.3 μM *S. cerevisiae*, IC_50_ = 60.3 μM Rat intestinal maltase, IC_50_ = 5.0 mM Rat instestinal sucrase, IC_50_ = 4.2 mM	*In vitro* assay	[142]
*G. elliptica*	2,4-dibromophenol	α-glucosidase inhibition against: *B. stearothermophilus*, IC_50_ = 230.3 μM *S. cerevisiae*, IC_50_ = 110.4 μM Rat intestinal maltase, IC_50_ = 4.8 mM Rat instestinal sucrase, IC_50_ = 3.6 mM	*In vitro* assay	[142]
*H. macroloba*	Water extract	α-glucosidase inhibition, IC_50_ = 6.388 mg/mL	*In vitro* assay	[132]
*I. okamurae*	DPHC	α-amylase inhibition, IC_50_ = 0.53 mM α-glucosidase inhibition, IC_50_ = 0.16 mM	*In vitro* assay	[143]
*L. japonica (rhizoids)*	BIP	α-glucosidase inhibition, IC_50_ = 38.00 μM	*In vitro* assay and hypoglycaemic effect in streptozotocin-induced diabetic mice	[144]
*A. nodosum*	Aqueous ethanolic extract	α-glucosidase inhibition, IC_50_ = 77 μg/mL	*In vitro* assay	[145]
*A. nodosum*	Water extract	α-amylase inhibition, IC_50_ = 1.34 μg phenolics α-glucosidase inhibition, IC_50_ = 0.24 μg phenolics	*In vitro* assay	[146]
*A. nodosum*	Phlorotannin-rich extract	α-amylase inhibition, IC_50_ ~0.1 μg/mL GAE α-glucosidase inhibition, IC_50_ ~20 μg/mL GAE	*In vitro* assay	[147]
*A. nodosum*	Cold water and ethanol extracts	α-amylase inhibition (water), IC_50_ = 53.6 μg/mL α-amylase inhibition (ethanol), IC_50_ = 44.7 μg/mL	*In vitro* assay	[148]
*F. vesiculosus*	Cold water and ethanol extracts	α-glucosidase inhibition (water), IC_50_ = 0.32 μg/mL α-glucosidase inhibition (ethanol), IC_50_ = 0.49 μg/mL	*In vitro* assay	[148]
*F. distichus*	Phlorotannin	α-amylase inhibition, IC_50_ = 13.9 μg/mL α-glucosidase inhibition, IC_50_ = 0.89 μg/mL	*In vitro* assay	[149]
*P. arborescens*	Methanolic extract	α-amylase inhibition, IC_50_ = 0.23 mg/mL α-glucosidase inhibition, IC_50_ = 0.26 mg/mL	*In vitro* assay	[150]
*S. paten*	DDBT	α-amylase inhibition, IC_50_ = 3.2 μg/mL α-glucosidase inhibition against: Rat intestinal maltase, IC_50_ = 114.0 μg/mL Rat intestinal sucrase, IC_50_ = 25.4 μg/mL	*In vitro* assay	[151]
*S. ringgoldianum*	Methanolic (80%) extract	α-amylase inhibition, IC_50_ = 0.18 mg/mL α-glucosidase inhibition, IC_50_ = 0.12 mg/mL	*In vitro* assay	[152]
*S. hemiphyllum*	Acetone extract	α-amylase inhibition, IC_50_ = 0.35 mg/mL Sucrase inhibition, IC_50_ = 1.89 mg/mL Maltase inhibition, IC_50_ = 0.09 mg/mL	*In vitro* assay	[153]

Kim and colleagues also purified BDDE but from red seaweed *Polyopes lancifolia* which inhibited the activities of α-glucosidase from *Bacillus stearothermophilus* (IC_50_ = 0.12 μM) and *S. cerevisiae* (IC_50_ = 0.098 μM), and was also effective against rat intestinal maltase (IC_50_ = 1.20 mM) and sucrase (IC_50_ = 1.00 mM) respectively [137]. Butyl-isobutylphthalate (BIP), a novel compound extracted from rhizoids of *Laminaria japonica* non-competitively inhibited α-glucosidase *in vitro* with IC_50_ value of 38.0 μM [144]. Furthermore, hypoglycaemic effect of BIP in streptozotocin-induced diabetic mice was attributed to the benzene-1,2-decarboxylate core in BIP [144]. A number of studies on *Ecklonia* also reported its inhibitory potential against carbohydrate hydrolytic enzymes. Methanolic and water extracts of brown seaweed *Ecklonia stolonifera* were found to possess α-glucosidase inhibitory activity against *Saccharomyces* and rat intestinal glucosidase [136]. The *E. stolonifera* methanolic extract exhibited stronger α-glucosidase inhibitory activity *in vitro* based on the IC_50_ values compared with the water extract and was attributed to higher polyphenolic content in the former which were estimated to be phlorotannins [136]. Furthermore, the IC_50_ values of *E. stolonifera* methanolic extract against rat intestinal maltase, sucrase, and glucoamylase were significantly lower compared with water extract against the same enzymes. Additionally, male KK-A^y^ mice fed with *E. stolonifera* diet for four weeks reduced lipid peroxidation and post-prandial blood glucose levels, suggesting strong antioxidant and anti-diabetic potential of *E. stolonifera* [136]. Several phlorotannins were isolated from *Ecklonia stolonifera* and *Eisenia bicyclis*, three of these phlorotannins were potent in inhibiting α-glucosidase activity *in vitro* [138]. Phlorofucofuroeckol-A showed the strongest inhibitory activity with IC_50_ value of 1.37 μM, followed by dieckol (IC_50_ = 1.61 μM) and 7-phloroeckol (IC_50_ = 6.13 μM), respectively. Dieckol is a competitive inhibitor of α-glucosidase, whereas phlorofucofuroeckol-A and 7-phloroeckol are non-competitive inhibitors of the same enzyme *in vitro* [138]. Similar phlorotannins isolated from *Ecklonia cava* were also previously examined by [139] and [140] for α-glucosidase and α-amylase inhibitory potential. The IC_50_ values for phlorofucofuroeckol-A, dieckol and 7-phloroeckol against α-glucosidase and α-amylase were higher in these earlier studies compared with those from *E. stolonifera* and *E. bicyclis* examined by [138]. Dieckol for example, was found to have different IC_50_ values for α-glucosidase inhibition in different studies with 10.8 μM [139], compared with 0.24 mM [140] and 1.61 μM [138] where these disparities may be attributed to differences in seasonal variation, species used and experimental procedures employed. *In vivo* study involving streptozotocin-induced diabetic mice revealed that dieckol from *E. cava* delayed carbohydrates absorption and reduced post-prandial blood glucose levels [140].

Phlorotannins from *Ecklonia maxima* were evaluated for their potential to inhibit α-glucosidase activity *in vitro* and eckol showed the best inhibitory activity (IC_50_ = 11.16 μM) compared with dibenzo[1,4]dioxine-2,4,7,9-tetraol (IC_50_ = 33.69 μM) and phloroglucinol (IC_50_ = 1991 μM) [141]. The inhibitory activity of eckol isolated from *E. maxima* [141] was stronger than that isolated from *E. stolonifera* and *E. bicyclis* [138] based on the IC_50_ values as shown in Table 1. Recently, a 12-week supplementation with dieckol-rich extract (1500 mg per day) from *Ecklonia cava* in a randomised, double-blind, placebo-controlled clinical trial involving 80 pre-diabetic individuals demonstrated significant reduction of insulin resistance and post-prandial hyperglycaemia. Furthermore, all haematological and biochemical parameters were normal with no significant adverse events observed during the supplementation period [154].

Kim and colleagues compared 10 seaweed species for α-glucosidase inhibitory activity and subsequently purified two bromophenols from red seaweed *Grateloupia elliptica* as the most potent among the species examined, which inhibited α-glucosidase from different organisms [142]. Inhibitory potential of 2,4,6-tribromophenol was more profound than 2,4-dibromophenol against *B. stearothermophilus* and *S. cerevisiae* α-glucosidase respectively, whereas 2,4-dibromophenol was relatively more potent against rat-intestinal maltase and sucrase but comparably weaker than acarbose and voglibose, possibly due to differences in substrate specificities [142]. Indeed, several bioactive compounds derived from seaweed exhibited significantly more inhibitory potential against *S. cerevisiae* α-glucosidase compared with the same enzyme from mammalian sources. These included BDDE from *S. latiuscula* [135], BDDE from *P. lancifolia* [137], bromophenols from *G. elliptica* [142] and extracts of *E. stolonifera* [136].

The inhibitory potential against carbohydrate carbolytic enzymes by brown seaweed *Ascophyllum nodosum* was also explored in several studies. The aqueous ethanolic extract of *A. nodosum* inhibited α-glucosidase with IC_50_ value of 77 μg/mL [145] whereas a recent study showed that cold water and ethanolic extracts of the same species inhibited α-amylase activity with IC_50_ values lower than 60 μg/mL [148]. Water extract of *A. nodosum* attenuated α-glucosidase (IC_50_ = 0.24 μg phenolics) and α-amylase (IC_50_ = 1.34 μg phenolics) activities *in vitro* and this was attributed to the high phenolic content [146]. Similarly, phlorotannin-rich extract of *A. nodosum* also inhibited α-glucosidase and α-amylase enzymes *in vitro*, which were also correlated to its phenolic content and was more effective compared with extracts of *Palmaria palmata* and *Alaria esculenta* [147]. *A. nodosum* extract showed inhibition of the enzymes with IC_50_ ~0.1 μg/mL GAE for α-amylase and IC_50_ ~20 μg/mL GAE for α-glucosidase. Furthermore, polyphenolic extract and enriched polyphenolic fraction from *A. nodosum* improved fasting blood glucose level in diabetic mice and decreased blood total cholesterol compared with the untreated group [145]. Other study focusing on seaweed polyphenols revealed that the high polyphenolic content (4.32 ± 0.74 μmol Gallic acid/g) in ethanolic extract of *Ulva rigida* may also confer anti-hyperglycaemic and antioxidant effects *in vivo* [155]. Additionally, *U. rigida* ethanolic extract significantly reduced blood glucose concentration in Wistar diabetic rats [155].

Cold water and ethanolic extracts of *Fucus vesiculosus* were also capable of inhibiting α-glucosidase activity *in vitro* with 50% activity inhibition at 0.32 μg/mL with water and 0.49 μg/mL with ethanolic extracts respectively [148]. Recently, crude extracts of *Fucus distichus* and *Alaria marginata* exhibited strongest carbohydrate hydrolysing enzymes inhibitory activity among 6 seaweed species examined [149]. *F. distichus* fractions were potent inhibitors of both α-glucosidase and α-amylase compared with acarbose, where IC_50_ values recorded at 0.89 and 13.9 μg/mL respectively and the inhibition was attributed to presence of phlorotannins in the fractions [149]. The methanolic extract of edible brown seaweed *Padina arborescens* inhibited the activities of α-glucosidase (IC_50_ = 0.26 mg/mL) and α-amylase (IC_50_ = 0.23 mg/mL) *in vitro* with stronger inhibitory effects compared with acarbose [150]. Additionally, *P. arborescens* extract was also found to significantly suppress post-prandial increase of blood glucose level in both normal and streptozotocin-induced diabetic mice possibly via delayed intestinal absorption of dietary carbohydrates [150].

Diphlorethohydroxycarmalol (DPHC) isolated from brown seaweed *Ishige okamurae* showed potent anti α-amylase (IC_50_ = 0.53 mM) and α-glucosidase (IC_50_ = 0.16 mM) activities which were higher than acarbose with no cytotoxicity observed [143]. Significant inhibition of post-prandial blood glucose increment and delayed absorption of dietary carbohydrates were also observed in DPHC-treated mice compared with normal and streptozotocin-induced diabetic mice [143].

Seaweeds of the *Sargassum* genus were also evaluated in several studies for their anti-diabetic properties. Phlorotannins in methanolic extract of *Sargassum ringgoldianum* were reported to inhibit the activities of both α-amylase (IC_50_ = 0.18 mg/mL) and α-glucosidase (IC_50_ = 0.12 mg/mL) *in vitro* [152]. These hypoglycaemic properties of *S. ringgoldianum* extract was also observed *in vivo* where lower blood glucose levels and delayed absorption of carbohydrates were observed in streptozotocin-induced diabetic mice compared with control [152]. A phloroglucinol derivative purified from the extract of *Sargassum paten*, 2-(4-(3,5-dihydroxyphenoxy)-3,5-dihydroxyphenoxy) benzene-1,3,5-triol (DDBT) significantly inhibited rat intestinal α-glucosidase as well as human salivary and pancreatic α-amylases [151]. DDBT was more potent against rat intestinal sucrase (IC_50_ = 25.4 μg/mL) compared with maltase (IC_50_ = 114.0 μg/mL). Furthermore, the α-amylase inhibitory activity of DDBT was competitive in nature and the IC_50_ value of 3.2 μg/mL is lower than several commercially available α-amylase inhibitors including acarbose [151]. Ethanolic and water extracts of *Sargassum polycystum* significantly reduced blood glucose, glycosylated haemoglobin (HbA1C), triglyceride and serum total cholesterol levels in streptozotocin-induced diabetic rat given high-sugar, high-fat diet [156]. Acetone extract of *Sargassum hemiphyllum* significantly inhibited α-amylase (IC_50_ = 0.35 mg/mL), maltase (IC_50_ = 0.09 mg/mL) and sucrase (IC_50_ = 1.89 mg/mL) activities *in vitro*, which was also attributed to the high concentrations of polyphenols (36.66 ± 2.01 mg/g) and fucoxanthin (15.12 ± 0.09 mg/g) [153]. Recently, our group reported that the crude water extracts of *Halimeda macroloba*, *Padina sulcata*, *Sargassum binderi* and *Turbinaria conoides* inhibited α-glucosidase activity *in vitro*, whereas the crude water extracts of *Laurencia snackeyi* and *Caulerpa lentillifera* showed no inhibition [132]. Furthermore, crude water extracts of *T. conoides* has broader activity by inhibiting both sucrase and amylase, and the preparation of these crude water extracts can be easily replicated in the domestic environment.

Generally, seaweed extracts and isolated compounds exhibited more inhibitory potency towards α-glucosidase activity compared with α-amylase in several studies. Compounds including fucofuroeckol A and dioxinodehydroeckol from *E. bicyclis* [134], DPHC from *I. okamurae* [143], dieckol from *E. cava* [139,140], phlorotannins from *F. distichus* [149] as well as extracts of *A. nodosum* [146] and *S. hemiphyllum* [153], which more profoundly inhibited α-glucosidase compared with α-amylase and this is desirable as excessive inhibition of α-amylase activity has been suggested to cause abnormal fermentation of undigested carbohydrates by the colonic microbiota. In contrast, DDBT from *S. paten* more significantly inhibited α-amylase than α-glucosidase activity [151].

### 3.2. Reduction of Glucose Levels via Miscellaneous Mechanisms

Reduction of glucose concentrations can also be achieved via different mechanisms using seaweed as listed in Table 2. In contrast to mammalian α-glucosidase, protein tyrosine phosphatase 1B (PTP1B) is a negative regulator of insulin signalling and is localised on the cytoplasmic surface of the endoplasmic reticulum in hepatic, muscular and adipose tissues [157]. The inhibition of α-glucosidase and PTP1B activities would lead to reduced plasma glucose levels and enhanced insulin action. Due to its ubiquity in the insulin-targeted tissues and its reported role in insulin resistance development [158], inhibiting PTP1B activity would be beneficial in treating T2DM. Phlorotannins isolated from edible brown seaweed *Ecklonia stolonifera* and *Eisenia* *bicyclis* significantly inhibited PTP1B activity [138]. Six phlorotannins were isolated and phlorofucofuroeckol-A was shown to be the most effective (IC_50_ = 0.56 μM) compared with phloroglucinol (IC_50_ = 55.48 μM). Bromophenol derivatives isolated from red seaweed *Rhodomela confervoides* inhibited PTP1B activity *in vitro* and showed hypoglycaemic effect in streptozotocin-induced diabetic Wistar rats [159]. The IC_50_ values for PTP1B inhibition of four bromophenol derivatives from *R. confervoides* were less than 2.5 μM with the strongest inhibitory activity shown by 2,2′,3-tribromo-3′,4,4′,5-tetrahydroxy-6′-ethyloxy-methyldiphenylmethane (IC_50_ = 0.84 μM) [159].

Bromophenols from red seaweed *Symphylocladia latiuscula* also showed strong inhibition of PTP1B activity with IC_50_ values ranging from 3.5 to 19.4 μM and 1,2-bis(2,3,6-tribromo-4,5-dihydroxyphenyl)-ethane was reported to be the most potent [160]. Strong inhibition of PTP1B activity was also observed in highly brominated metabolites from another species of red seaweed, *Laurencia similis* [161]. Two brominated compounds, 2′,5′,6′,5,6-pentabromo-3′,4′,3,4-tetramethoxybenzo-phenone and 3′,5′,6′,6-tetrabromo-2,4-dimethyldiphenyl ether, inhibited PTP1B activity *in vitro* with IC_50_ values of 2.66 μg/mL and 2.97 μg/mL respectively [161].

**Table 2:** Glucose levels reduction by seaweed via miscellaneous mechanisms.

Seaweed	Active Agent(s)	Activity	Test System(s)	References
*E. bicyclis*	Phloroglucinol	AGE formation inhibition at 1 mM: Eckol, inhibition = 96.2% 1-(3′,5′-dihydroxyphenoxy)-7-(2″,4″,6″-trihydroxyphenoxy)-2,4,9-trihydroxydibenzo-1,4-Dioxin, inhibition = 91.1% Dieckol, inhibition = 86.7%	*In vitro* assay	[133]
*L. japonica*	Porphyrin derivatives	AGE formation inhibition by: Pheophorbide a, IC_50_ = 49.43 μM Pheophytin a, IC_50_ = 228.71 μM	*In vitro* assay	[162]
*L. japonica*	Porphyrin derivatives	Aldose reductase inhibition by: Pheophorbide a, IC_50_ = 12.31 μM Pheophytin a, IC_50_ ˃ 100 μM	Rat lens aldose reductase assay *in vitro*	[162]
*E. stolonifera*	Phloroglucinol derivatives	Aldose reductase inhibition by: Dioxinodehydroeckol, IC_50_ = 21.95 μM 7-Phloroeckol, IC_50_ = 27.54 μM Dieckol, IC_50_ = 42.39 μM Eckol, IC_50_ = 54.68 μM Phloroglucinol, IC_50_ = 72.54 μM Phlorofucofuroeckol-A, IC_50_ = 125.45 μM	Rat lens aldose reductase assay *in vitro*	[163]
*C. fulvescens*	Capsofulvesin A Capsofulvesin B Chalinasterol	Aldose reductase inhibition by: Capsofulvesin A, IC_50_ = 52.53 μM Capsofulvesin B, IC_50_ = 101.92 μM Chalinasterol, IC_50_ = 345.27 μM	Rat lens aldose reductase and advanced glycation end-products inhibition assays *in vitro*	[164]
*E. cava*	Methanolic extract	Reduction of post-prandial blood glucose level partially attributed to the AMP-activated protein kinase/ACC and PI-3K/Akt cellular signal pathways	C_2_C_12_ myoblast cells and streptozotocin-induced diabetic mice	[165]
*S. binderi*	Ethanolic precipitates	DPP-4 inhibition, IC_50_ = 2.194 mg/mL	*In vitro* assay	[132]
*P. sulcata*	Ethanolic precipitates	DPP-4 inhibition, IC_50_ = 2.306 mg/mL	*In vitro* assay	[132]
*T. conoides*	Ethanolic precipitates	DPP-4 inhibition, IC_50_ = 3.594 mg/mL	*In vitro* assay	[132]
*S. binderi*	Water extracts	Stimulation of GIP secretion of 5.46 pM GIP per million cells per h at 2.5 mg/mL	pGIP/neo STC-1 cells *in vitro*	[132]
*P. sulcata*	Water extracts	Stimulation of GIP secretion of 4.92 pM GIP per million cells per h at 10.0 mg/mL	pGIP/neo STC-1 cells *in vitro*	[132]
*T. conoides*	Water extracts	Stimulation of GIP secretion of 5.00 pM GIP per million cells per h at 2.5 mg/mL	pGIP/neo STC-1 cells *in vitro*	[132]
*S. binderi*	Butanol fraction	Stimulation of GLP-1 secretion of 56.38 pM GIP per million cells per h at 5.0 mg/mL	pGIP/neo STC-1 cells *in vitro*	[132]
*P. sulcata*	Butanol fraction	Stimulation of GLP-1 secretion of 40.67 pM GIP per million cells per h at 5.0 mg/mL	pGIP/neo STC-1 cells *in vitro*	[132]
*I. foliacea*	Octaphlorethol A	Increased glucose uptake by GLUT4 via PI3-K/Akt and AMPK signalling pathways	L6 rat myoblast cells *in vitro*	[166]
*A. nodosum*	Aqueous ethanolic extract	Stimulation of basal glucose uptake into cells	3T3-L1 adipocytes in *vitro*	[145]
*U. pinnatifida*	Fucoxanthin	Promotion of Adrb3 and GLUT4 mRNA expressions in skeletal muscle tissues	High-fat diet mice	[167]
*P. binghamiae*	Extract	Increased up-regulation of GLUT4 mRNA, PPARγ and terminal marker protein aP2 up-regulation Stimulation of 3T3-L1 adipocytes differentiation and expression of IRS-1 with increased uptake of glucose	3T3-L1 adipocytes *in vitro*	[168]
*L. similis*	Bromophenols	PTP1B inhibition by: 2′,5′,6′,5,6-pentabromo-3′,4′,3,4-tetramethoxybenzo-phenone, IC_50_ = 2.66 μg/mL 3′,5′,6′,6-tetrabromo-2,4-dimethyldiphenyl ether, IC_50_ = 2.97 μg/mL 2,5,8-tribromo-3-bromoamino-7-bromomethylnaphthalene, IC_50_ = 65.30 μg/mL 2,5,6-tribromo-3-bromoamino-7-bromomethylnaphthalene, IC_50_ = 69.80 μg/mL	*In vitro* assay	[161]
*R. confervoides*	Bromophenol derivatives	PTP1B inhibition by: 2,2′,3-tribromo-3′,4,4′,5-tetrahydroxy-6′-ethyloxy-methyldiphenylmethane, IC_50_ = 0.84 μM Bis(2,3-dibromo-4,5-dihydroxybenzyl)ether, IC_50_ = 1.5 μM 3-bromo-4,5-bis(2,3-dibromo-4,5-dihydroxybenzyl)pyrocatechol, IC_50_ = 1.7 μM 2,2′,3,3′-tetrabromo-4,4′,5,5′-tetra-hydroxydiphenyl methane, IC_50_ = 2.4 μM	PTP1B and hypoglycaemic effect in streptozotocin-induced diabetic Wistar rats	[159]
*E. stolonifera* *E. bicyclis*	Phlorofucofuroeckol-A Dieckol 7-Phloroeckol Eckol Dioxinodehydroeckol Phloroglucinol	PTP1B inhibition by: Phlorofucofuroeckol-A, IC_50_ = 0.56 μM Dieckol, IC_50_ = 1.18 μM 7-Phloroeckol, IC_50_ = 2.09 μM Eckol, IC_50_ = 2.64 μM Dioxinodehydroeckol, IC_50_ = 29.97 μM Phloroglucinol, IC_50_ = 55.48 μM	*In vitro* assay	[138]
*S. latiuscula*	Bromophenols	PTP1B inhibition by: 1,2-bis(2,3,6-tribromo-4,5-dihydroxyphenyl)-ethane, IC_50_ = 3.5 μM 2,3,6-tribromo-4,5-dihydroxybenzyl methyl ether, IC_50_ = 3.9 μM Bis(2,3,6-tribromo-4,5-dihydroxyphenyl)-ethane, IC_50_ = 4.3 μM 2,3,6-tribromo-4,5-dihydroxybenzaldehyde, IC_50_ = 19.40 μM	*In vitro* assay	[160]
*E. cava*	Methanolic extract	Decreased blood glucose concentration Prevented the loss of β-cell mass hence improved insulin secretion	Streptozotocin-induced diabetic mice	[169]
*E. cava*	Dieckol	Reduction of blood glucose, glycosylated hemoglobin levels Reduction of hepatic lipids concentration and also improvement of impaired glucose tolerance Reduction of glucose-6-phosphatase and phosphoenolpyruvate carboxykinase enzymes activities Increased glucokinase activity	C57BL/KsJ-db/db diabetic mice	[170]
*H. elongata*	Crude polysaccharides	Reduction of post-prandial blood glucose level	Alloxan-induced diabetic rabbits	[96]
*H. elongata*	Fucan	Reduction of post-prandial blood glucose level	Alloxan-induced diabetic rabbits	[97]
*E. stolonifera*	Polyphenol-rich methanolic extract (Phlorotannins)	Reduction of post-prandial blood glucose and lipid peroxidation levels	Male diabetic KK-A^y^ mice	[136]
*E. cava*	Dieckol	Reduction of post-prandial blood glucose level and delayed absorption of dietary carbohydrates	Streptozotocin-induced diabetic mice	[140]
*A. nodosum*	Polyphenolic extracts	Improved fasting blood glucose level Decreased blood total cholesterol and glycated serum protein levels	Streptozotocin-induced diabetic mice	[145]
*U. rigida*	Ethanolic extract	Reduction of post-prandial blood glucose level Antioxidant activity	Wistar diabetic rats	[155]
*I. okamurae*	DPHC	Reduction of post-prandial blood glucose level and delayed absorption of dietary carbohydrates	Streptozotocin-induced diabetic mice	[143]
*P. arborescens*	Methanolic extract	Reduction of post-prandial blood glucose level delayed absorption of dietary carbohydrates	Streptozotocin-induced diabetic mice	[150]
*S. ringgoldianum*	Methanolic (80%) extract	Reduction of post-prandial blood glucose level and delayed absorption of dietary carbohydrates	Streptozotocin-induced diabetic mice	[152]
*S. polycystum*	Ethanolic and water extracts	150 and 300 mg/kg of ethanolic extract and 300 mg/kg of water extract significantly reduced blood glucose and HbA1C levels Significant reduction of serum total cholesterol, triglyceride levels	Streptozotocin-induced diabetic rat given high-sugar, high-fat diet	[156]

The inhibition of AGEs formation and aldose reductase activity is another approach currently being explored in managing hyperglycaemia using seaweed. Aldose reductase is a crucial rate-controlling enzyme in the polyol pathway implicated in the pathogenesis of various diabetes-related vascular complications due to increased sorbitol and ROS concentrations [171,172,173]. Hyperactivation of the polyol pathway due to hyperglycaemia lead to accumulation of AGEs in tissues, contributing to development of complications such as retinopathy and nephropathy. Genetic deletion of aldose reductase gene in mutant diabetic C57BI/6 mice inhibited superoxide production in retina and substantially attenuated hyperglycaemia-induced degeneration of retinal capillaries [174]. Several phloroglucinols at 1 mM inhibited formation of AGE *in vitro*, where eckol showed the greatest inhibition at 96.2% [133]. Similarly, phloroglucinol derivatives extracted from *E. stolonifera* inhibited aldose reductase activity *in vitro* with dioxinodehydroeckol being the most active (IC_50_ = 21.95 μM) compared with phlorofucofuroeckol-A (IC_50_ = 125.45 μM) [163]. The extracts of edible green seaweed *Capsosiphon fulvescens* were found to inhibit aldose reductase activity and out of 11 compounds identified from *C. fulvescens*, only capsofulvesin A, capsofulvesin B as well as chalinasterol exhibited significant inhibition of aldose reductase activity at IC_50_ values of 52.53 μM, 101.92 μM, and 345.27 μM respectively, but no significant inhibition of AGE formation was observed [164]. In contrast, pheophorbide A isolated from *Laminaria japonica* possesses greater inhibition towards aldose reductase activity (IC_50_ = 12.31 μM) compared with attenuating AGE formation (IC_50_ = 49.43 μM) *in vitro* and these inhibitory activities are relatively stronger compared with pheophytin A [162]. Therefore, inhibition of PTP1B and aldose reductase activities is also a possible avenue where seaweed can play a beneficial role in diabetes management.

Incretin hormones are insulinotropic intestinal hormones which stimulate secretion of insulin in a glucose-dependent manner and two known incretin hormones are glucose-dependent insulinotrophic polypeptide (GIP) and glucagon-like peptide-1 (GLP-1). These intestinal hormones are rapidly broken down by dipeptidyl-peptidase-4 (DPP-4) enzyme, effectively removing their ability to stimulate insulin secretion and reduce blood glucose levels [175,176]. Furthermore, incretin hormones are known to promote pancreatic β-cell proliferation and inhibit apoptosis, which can increase β-cell mass and intensify production of insulin [177]. Increasing GIP and GLP-1 secretion and inhibiting the activity of DPP-4 are a proven pharmacological strategy for controlling hyperglycaemia in T2DM [178]. Our group recently reported for the first time that brown seaweed species namely *Sargassum binderi*, *Padina sulcata* and *Turbinaria conoides* significantly inhibited DPP-4 activity *in vitro* with IC_50_ values of 2.194, 2.306 and 3.594 mg/mL for their ethanolic precipitates respectively [132]. Furthermore, the crude water extracts of these brown seaweeds also stimulated GIP secretion from pGIP/neo STC-1 cells *in vitro* with *S. binderi* elicited the greatest secretion of 5.46 pM GIP per million cells per h at 2.5 mg/mL of *S. binderi* water extracts, followed by *T. conoides* and *P. sulcata*. Additionally, 5 mg/mL of *S. binderi* and *P. sulcata* butanol fraction were also found to stimulate GLP-1 secretion from pGIP/neo STC-1 cells at 56.38 and 40.67 pM GIP per million cells per h, respectively [132].

Skeletal muscle tissues are the primary tissue for glucose uptake and disposal, accounting for approximately 70% of glucose uptake from blood [179], hence its important role in energy balance regulation [180]. Insulin-stimulated uptake of glucose into skeletal muscle tissues is crucial in glycaemia management [181] and this process can be activated in at least two major pathways namely the phosphoinositide 3-kinase/Akt (PI3K/Akt) and 5′-adenosine monophosphate-activated protein kinase (AMPK) pathways. PI3K and Akt activation promotes GLUT4 translocation to the plasma membrane [182] whereas AMPK activation accelerates adenosine triphosphate (ATP) production via catabolic pathway involving uptake of glucose and fatty acid oxidation [181]. Physical activities trigger AMPK in skeletal muscle tissues leading to increased uptake of glucose by muscle cells. Furthermore, past studies have also demonstrated that AMPK activation can also be achieved by various compounds, including hispidulin [183], theaflavin, and epigallocatechin gallate, from tea leaves [184], as well as T2DM medications, such as thiazolidinediones [185] and metformin [186]. Hence, bioactive compounds from seaweed, which can promote glucose uptake into cells will be useful in T2DM management. Different seaweed species have been examined for their ability to induce glucose uptake into cells and tissues, with the aim of reducing circulating glucose concentrations and hyperglycaemia. The aqueous ethanolic extract of *A. nodosum* stimulated the basal glucose uptake into 3T3-L1 adipocytes *in vitro* by approximately three-fold [145]. In another study on 3T3-L1 adipocytes, extracts from *Petalonia binghamiae* stimulated the up-regulation of GLUT-4 mRNA, peroxisome proliferator-activated receptor-γ (PPARγ) and terminal marker protein aP2 [168]. PPARγ which are expressed in numerous tissues especially in white and brown adipose tissues plays a crucial role in adipogenesis regulation as well as lipid and glucose metabolism. It is a recognised pharmacological target for glitazones which indirectly increase insulin-stimulated glucose uptake by skeletal muscle tissues, hepatocytes and adipocytes [187]. *P. binghamiae* extract also induced 3T3-L1 adipocytes differentiation and stimulated the expression of insulin receptor substrate-1 (IRS-1) with concomitant increase of glucose uptake by the adipocytes *in vitro* [168]. Mice which were subjected to high-fat diet and subsequently given fucoxanthin isolated from *Undaria pinnatifida*, showed elevated β3-adrenergic receptor (Adrb3) and GLUT-4 mRNA expressions in skeletal muscle tissues [167]. Increased Adrb3 and GLUT-4 activities enhance lipolysis in adipose tissue and glucose uptake into cells, highlighting the anti-diabetic benefit of fucoxanthin. Post-prandial blood glucose concentration in streptozotocin-induced diabetic mice was reduced by *Ecklonia cava* methanolic extract and the observed reduction was partially attributed to the AMPK/ACC and PI3K/Akt cellular signalling pathways as studied in C_2_C_12_ myoblast cells [165]. The increased uptake of glucose by L6 myoblast cells *in vitro* induced by octaphlorethol A extracted from *Ishige foliacea* was also suggested to involve the AMPK and PI3K/Akt signalling pathways and increased GLUT4 translocation to the plasma membrane, hence aiding in glucose uptake process [166].

### 3.3. Anti-Obesity and Anti-Inflammatory Properties of Seaweed

Metabolic overload due to over-nutrition and excess caloric intake can lead to hyperglycaemia as well as obesity. Excessive levels of blood glucose and free fatty acids impose a stressful condition for pancreatic β-cells and other insulin-sensitive cells including adipose tissues, culminating in the local secretion of cytokines- and chemokines-causing inflammation. These pro-inflammatory factors promote further recruitment of immune cells, contributing to an inflammatory environment. Release of pro-inflammatory factors into the systemic circulation causes inflammatory response in other tissues including pancreatic β-islets. Inflammation in target tissues induced by hyperglycaemia and obesity significantly increases the risk of insulin resistance and β-cell failure, leading to the development of T2DM.

Although some seaweeds are constantly exposed to an environment with high oxygen concentrations and light availability, they are not normally affected by serious photodynamic damage during metabolism as they possess a myriad of antioxidative mechanisms and antioxidants in the form of pigments and polyphenols [188]. The antioxidant properties of various crude extracts and compounds from brown seaweed have been recently reviewed [189], hence we only concentrated on previous studies which examined anti-inflammatory benefit of seaweed in diabetes setting. Seaweed bioactive compounds were found to act either directly or indirectly on various targets of oxidative stress and inflammatory response.

Hyperglycaemia can lead to glucose auto-oxidation, protein glycation, as well as polyol metabolism activation that collectively accelerate ROS production resulting in oxidative chemical alterations of DNA, proteins and lipids in various tissues [190,191,192]. Furthermore, glucose auto-oxidation can potentially cause functional damage to proteins via changes to important arginine residues [193]. The elevated generation of mitochondrial superoxide due to hyperglycaemia is accompanied by increased nitric oxide production [194] which promotes the production of other ROS-causing, oxidative stress and inflammatory responses. This leads to insulin resistance primarily in skeletal muscle tissues, pancreatic β-cells dysfunction and reduced insulin secretion [195,196]. Excessive oxidative stress induced by hyperglycaemia is also implicated in the development of other diabetes-related health problems including macro- and microvascular complications.

Table 3 lists anti-obesity and anti-inflammatory properties of seaweed, which are potentially beneficial in managing diabetes. Dieckol and enzymatic digest obtained from *Ecklonia cava* were shown to be protective against hyperglycaemia-induced oxidative stress [197,198]. Dieckol administered at 10 or 50 μg/mL and the *E. cava* enzymatic digest at 10 or 100 μg/mL reduced cellular oxidative damage caused by high glucose concentration to the human umbilical vein endothelial cells *in vitro*. Furthermore, ROS production was reduced and cellular over-expression of inflammation-associated proteins including nuclear factor-kappa B (NF-κB), cyclooxygenase-2 (COX-2) and inducible nitric oxide synthase (iNOS) decreased in the presence of the *E. cava* enzymatic digest [197] and dieckol [198] suggesting their anti-inflammatory and cellular protective properties against glucotoxicity of hyperglycaemia.

The methanolic extract of *Ecklonia stolonifera* which contains high level of polyphenols suppressed lipid peroxidation and oxidative stress in diabetic KK-A^y^ mice [136]. Formation of lipid peroxides due to the presence of ROS lead to a chain reaction in oxidative stress and damages various other biomolecules resulting in cell death [199]. Therefore, attenuating high concentrations of ROS and reducing the risk of lipid peroxidation are important considering that malondialdehyde, the product of lipid peroxidation has been detected at elevated levels in diabetic patients [200].

Hot water extract of *Ascophyllum nodosum* showed a dose-dependent free-radical scavenging activity *in vitro*, with 70% inhibition reported at 25 mg/mL and this antioxidant activity was attributed to its high content of polyphenols [146]. Eckol, dibenzo[1,4]dioxine-2,4,7,9-tetraol and phloroglucinol from *Ecklonia maxima* also showed free-radical scavenging activity as measured using the DPPH assay and the EC_50_ values were recorded as 0.008 μM, 0.012 μM, and 0.128 μM, respectively [141]. These studies demonstrated the seaweed species examined were potentially useful in reducing lipid peroxidation and excessive ROS concentrations via its antioxidant and radical-scavenging properties.

**Table 3:** Anti-obesity and anti-inflammatory properties of seaweed.

Seaweed	Active Agent(s)	Activity	Test System(s)	References
*E. stolonifera*	Polyphenol-rich methanolic extract	Suppression of lipid peroxidation and oxidative stress	Male diabetic KK-A^y^ mice	[136]
*E. maxima*	Eckol Dibenzo [1,4] dioxine-2,4,7,9-tetraol Phloroglucinol	Free-radical scavenging activity by: Eckol, EC_50_ = 0.008 μM Dibenzo[1,4]dioxine-2,4,7,9-tetraol, EC_50_ = 0.012 μM Phloroglucinol, EC_50_ = 0.128 μM	DPPH assay *in vitro*	[141]
*E. cava*	Methanolic extract	Decreased triglyceride and total cholesterol concentration	Streptozotocin-induced diabetic mice	[169]
*E. cava*	Dieckol	Pancreatic lipase inhibition with IC_50_ = 0.26 mg/mL	Pancreatic lipase inhibitory assay *in vitro*	[201]
*E. bicyclis*	Phloroglucinol derivatives (phlorotannins)	Pancreatic lipase inhibition by: 7-phloroeckol, IC_50_ = 12.7 μM Fucofuroeckol A, IC_50_ = 37.2 μM Eckol, IC_50_ = 76.6 μM Dieckol, IC_50_ = 99.3 μM	Pancreatic lipase inhibitory assay *in vitro*	[202]
*A. nodosum*	Water extract	Dose-dependent free-radical scavenging activity, 70% inhibition at 25 mg/mL	DPPH assay *in vitro*	[146]
*I. okamurae*	Ethanolic extract	Inactivation of pro-inflammatory NF-κB transcription factor Suppression of pro-inflammatoryTNF-α, IL-6, IL-1β and PEG2 expressions	Macrophages *in vitro*	[203]
*I. foliacea*	Octaphlorethol A	Increased anti-oxidative GSH-px, CAT and SOD enzymes activities Reduction of ROS over-production Cytoprotection of pancreatic β-cells	RINm5F rat insulinoma cells *in vitro*	[204]
*E. cava*	Enzymatic digest	Anti-oxidative effect at 10 or 100 μg/mL Reduction of ROS over-production Reduction of pro-inflammatory NF-κB, COX-2 and iNOS expressions	Human umbilical vein endothelial cells *in vitro*	[197]
*E. cava*	Enzymatic hydrolysate	Increased anti-oxidative GSH-px, CAT and SOD enzymes activities Inhibition of ROS and nitric oxide over-production	INS-1 pancreatic β-cells *in vitro*	[205]
*E. cava*	Dieckol	Anti-oxidative effect at 10 or 50 μg/mL Reduction of ROS over-production Reduction of pro-inflammatory NF-κB, COX-2 and iNOS expressions	Human umbilical vein endothelial cells *in vitro*	[198]
*E. cava*	Dieckol	Reduction of oxidative stress *in vivo* by increasing the activities of hepatic antioxidant enzymes including glutathione peroxidase and superoxide dismutase	db/db diabetic mice	[206]
*U. pinnatifida*	Fucoxanthin	Suppression of adipose tissue weight gain via up-regulation of UCP-1expression Increased hepatic DHA level	Male Wistar rats and female KK-A^y^ mice	[207,208]
*U. pinnatifida*	Fucoxanthin	Suppression of adipose tissue and body weight gain Reduction of MCP-1 mRNA expressions	High-fat diet mice	[167]
*U. pinnatifida*	Fucoxanthin	Down-regulation of pro-inflammatory cytokines expressions *in vivo* and *in vitro* Reduction of pro-inflammatory MCP-1, PAI-1, IL-6 and TNF-α mRNA expressions Suppression of white adipose tissue weight gain	Diabetic KK-A^y^ mice	[209]
*U. pinnatifida*	Fucoxanthinol	Reduction of pro-inflammatory iNOS and COX-2 mRNA over-expression Reduction of pro-inflammatory MCP-1 and IL-6 mRNA over-expression in cells	RAW264.7 macrophage-like cells and 3T3-F442A adipocytes *in vitro*	[209]
*P. binghamiae*	Ethanolic and water extracts	Decreased adiposity and weight-gain	Obese and diabetic mice	[168,210]
*P. binghamiae*	Fucoxanthin	Reduction of body and adipose tissue weight Reduction of lipogenesis and promotion of β-oxidation	High fat-diet mice	[211]

Methanolic extract of *Ecklonia cava* prevented the loss of β-cell mass leading to increased production of insulin [169] whereas octaphlorethol A, a phenolic compound isolated from *Ishige foliacea* protected pancreatic β-cells damage caused by hyperglycaemia due to streptozotocin treatment [204]. Cellular damage and apoptosis of RINm5F rat insulinoma cells treated with streptozotocin were decreased with *in vitro* pre-treatment of octaphlorethol A at 12.5 μg/mL or 50 μg/mL where dose-dependent reduction of ROS was observed [204]. Cytoprotective effect of octaphlorethol A against cell damage was attributed to the increased activities of antioxidative enzymes such as glutathione peroxidase (GSH-px), catalase (CAT) and superoxide dismutase (SOD) [204]. Stimulation of antioxidant enzymes activities increased insulin levels due to the cytoprotective effect, suggesting the cellular viability was not affected by the excessive oxidative stress in the presence of octaphlorethol A thereby ensuring continuous insulin production [204]. Other studies also demonstrated seaweed potential in stimulating hepatic antioxidative enzymes activities. Enzymatic hydrolysate from *Ecklonia cava* prevented cell damage caused by glucotoxicity via increased antioxidant enzymes activities including GSH-px, CAT and SOD in INS-1 pancreatic β-cells *in vitro* [205]. The over-production of ROS and nitric oxide due to high glucose pre-treatment of INS-1 pancreatic β-cells were inhibited in a dose-dependent manner by *E. cava* enzymatic hydrolysate [205]. Dieckol isolated from *Ecklonia cava* also reduced oxidative stress *in vivo* by increasing hepatic antioxidant enzymes activities including GSH-px and SOD in db/db diabetic mice [206]. The production of ROS and the levels of GSH-px and mitochondrial manganese superoxide dismutase (MnSOD) expression were also observed to be significantly lower in the presence of fucoidan [123]. Seaweed supplementation consisting of *Undaria pinnatifida* and *Saccharina japonica* with 48 g total daily intake for four weeks by T2DM patients significantly stimulated antioxidant enzymes GSH-px and CAT, as well as reduced the level of thiobarbituric acid reactive substances (TBARS) in erythrocytes [86]. Furthermore, the extracts and MUFA derivatives isolated from the green seaweed *Ulva lactuca* induced many ARE-driven antioxidant genes in various mouse tissues *in vivo* which revealed its cytoprotective property [58]. Therefore, seaweed bioactive compounds are capable of inducing elevated activities of various antioxidative enzymes to counter ROS over-production, effectively preventing deterioration of β-cells due to oxidative damage. Ensuring the viability of pancreatic β-cells allows uninterrupted production of insulin. The current data show some promising results. Furthermore, the effect of seaweed bioactive compounds in promoting regeneration of damaged pancreatic β-cell islets is another promising avenue but currently remains unexplored.

As inflammation is associated not only with hyperglycaemic condition but also obesity, several studies demonstrated anti-obesity effect of seaweed consumption suppressed adipose tissue weight gain and attenuated pro-inflammatory cytokines. Obesity is a condition where excessive accumulation of lipid in adipose tissue poses adverse effects to health, including increasing the risk of diabetes and other associated co-morbidities. In 2014, 1.9 billion adults worldwide were estimated to be overweight where 600 million were considered obese with an elevated risk of developing diabetes [212]. It was demonstrated that individuals who are overweight and gained 1 kg of weight annually for a 10-year period, had nearly 50% increased risk of developing diabetes in the next 10 years compared with normal individuals [213].

The main functions of adipose tissues include body insulation, protection and as a depository for post-prandial free fatty acids that can be released later during caloric need. Phlorotannins from several seaweed species have been shown to possess lipase inhibitory activity. Several phloroglucinol derivatives isolated from *Eisenia bicyclis* inhibited pancreatic lipase activity *in vitro* with 7-phloroeckol (IC_50_ = 12.7 μM) showing the strongest inhibition [202] similarly with dieckol from *Ecklonia cava* which also exhibited pancreatic lipase inhibitory activity [201] but with lower IC_50_ value of 0.26 mg/mL. Apart from storing excess calories as lipids, adipose tissue has also been recognised as an important source of cytokines. Obesity is associated with chronic, low inflammatory condition due to alterations in macrophages and adipose tissue functions [214], obese adipose tissue has a role in increasing the risk of developing insulin resistance in peripheral tissues [215,216,217]. Insulin resistance was known to be associated with obesity where free fatty acids was shown to serve as important endocrine factor in regulating metabolic function regulation in tissues and the subsequent accumulation of macrophages leads to pro-inflammatory condition in obese adipose tissues [218,219]. Therefore, being overweight and obese increases the risk of developing diabetes due to low chronic inflammatory conditions involving adiposity, contributing to the development of insulin resistance. Controlling unhealthy weight gain and attenuating obesity-related inflammation via seaweed consumption may be a viable strategy in T2DM management.

A xanthophyll (carotenoid) derived from *Undaria pinnatifida,* fucoxanthin was found to significantly reduce the weight of white adipose tissue in diabetic mice by up-regulating the expression of mitochondrial uncoupling protein-1 (UCP1), which is an important factor in metabolic thermogenesis to prevent excess accumulation of fat [207,208] and also significantly elevated hepatic docosahexaenoic acid (DHA) which is an important PUFA [208]. As UCP-1 plays a crucial role in increasing energy expenditure, heat-generation and reducing over-production of ROS particularly in brown adipose tissue, harnessing UCP-1 to increase thermogenesis via tissue-specific UCP-1 as well as glucose consumption during high-energy intake may be a useful route to reduce weight gain and diabetic risk. Furthermore, mice fed with high-fat diet for 10 weeks exhibited signs of hyperglycaemia, hyperinsulinemia and hyperleptinemia as well as increased mRNA expression for monocyte chemoattractant protein-1 (MCP-1) [167]. The introduction of fucoxanthin-rich lipid from *Undaria pinnatifida* normalised diabetes and obesity-related parameters. High-fat diet mice that consumed fucoxanthin rich-wakame lipids showed significant suppression of white adipose tissue and body weight gain, promotion of Adrb3 and GLUT4 mRNA expressions in both white adipose and skeletal adipose tissues. Furthermore, MCP-1 mRNA expression was also reduced [167].

Inflammation due to obesity also involved increased levels of pro-inflammatory cytokines such as TNF-α, MCP-1 and interleukin-6 (IL-6). Indeed, the elevated levels of MCP-1 and plasminogen activator inhibitor-1 (PAI-1) in obese adipose tissues are involved in different aspects of adipose cells development [195,219,220]. TNF-α inhibits the expression of genes which are involved in adipose cells differentiation and those important in insulin signalling cascade [221], therefore, it is considered as an important cytokine linked to decreased insulin sensitivity and is found to be elevated in serum of obese patients [222,223]. Pro-inflammatory cytokines, such as IL-6 and TNF-α, released from obese adipose tissue were primarily the product of localised immune cells, such as macrophages, which were recruited to these tissues [218,219]. These macrophages, being the most abundant type of immune cells present, produce a myriad of cytokines involved in chemotaxis such as MCP-1 and recruiting additional immune cells to the sites hence promoting a cycle of pro-inflammatory environment. Indeed, the majority of cytokines produced due to obesity are secreted by macrophages localised in obese adipose tissues [218,219] and reduced number of macrophages corresponds to both weight-loss [224] and degree of insulin resistance [225].

Methanolic extracts of an edible species of brown seaweed that is also popular in Korean traditional medicine, *Undaria pinnatifida* and a species of green seaweed, *Ulva linza* were observed to inhibit inflammatory response, which was attributed to their PUFA content [80]. Fucoxanthin and its dietary metabolite, fucoxanthinol down-regulated the expression of pro-inflammatory cytokines *in vivo* and *in vitro* [209]. Fucoxanthin reduced the mRNA expression of MCP-1, PAI-1, IL-6 and TNF-α in white adipose tissue of diabetic KK-A^y^ mice. Fucoxanthinol reduced iNOS and COX-2 mRNA over-expression in RAW264.7 macrophage-like cells, as well as reduction of MCP-1 and IL-6 mRNA over-expression in differentiating 3T3-F442A adipocytes *in vitro* [209]. Fucoidan from *U. pinnatifida* also reduced the expression of MCP-1, PAI- and TNF-α during adipogenesis in 3T3-L1 cells *in vitro* [124]. High levels of pro-inflammatory cytokines such as PAI-1, IL-6, TNF-α and reduced secretion of anti-inflammatory IL-10 are associated with increased insulin resistance [196,226,227,228]. The suppression of white adipose tissue weight gain and the reduction of pro-inflammatory cytokines in diabetic obese KK-A^y^ mice and cells *in vitro* were suggested as possible mechanisms behind the anti-diabetic and anti-obesity properties of edible *U. pinnatifida* [209]. Reduction of body weight and adipose tissue weight gain were also observed in obese mice given *Petalonia binghamiae* extract at 150 mg/kg/day for 70 days [211]. Previously, ethanolic and water extracts of *P. binghamiae* exhibited anti-diabetic effects as well as decreased adiposity and weight-gain in diabetic and obese mice [168,210]. It was suggested that *P. binghamiae* extract or fucoxanthin improves obesity in high-fat diet mice via lipogenesis reduction and promotion of β-oxidation [211].

Furthermore, as the depository of excess energy, adipose tissue is capable of releasing free fatty acids via triglyceride lipolysis during caloric need [229] and some free fatty acids can potentially induce inflammatory response in cells. Both high concentrations of TNF-α and free fatty acids produced by obese adipose tissue play a crucial role in the pathogenesis of insulin resistance [230]. Insulin resistance in combination with high-energy food consumption, prevents adipose tissue from inhibiting lipolysis leading to increased free fatty acid levels [231]. Ethanolic extract of *Ishige okamurae* inhibited oxidative stress by inactivating NF-κB transcription factor in macrophage [203]. Production of inflammatory cytokines whose expression are regulated by NF-κB, such as TNF-α, IL-6, interleukin-1β (IL-1β) and prostaglandin E2 (PEG2) in macrophage was suppressed *in vitro* upon treatment with *I. okamurae* ethanolic extract [203]. Methanolic extract of *Ecklonia cava* decreased blood glucose, triglyceride and total cholesterol concentrations in streptozotocin-induced diabetic mice [169]. Diabetic C57BL/KsJ-db/db mice consuming dieckol-rich diet containing extract of *Ecklonia cava* exhibited lower blood glucose, HbA1C, hepatic lipids concentrations and also improvement of impaired glucose tolerance. Furthermore, increased glucokinase activity with concomitant reduction of glucose-6-phosphatase and phosphoenolpyruvate carboxykinase enzymes activities were observed in mice given the dieckol-rich diet [170].

### 3.4. Differences in the Potency of Seaweed Bioactive Compounds against Diabetic Targets

Differences in the potency of various extracts and bioactive compounds derived from seaweed for different targets discussed in this review can be attributed to several factors including environmental and seasonal variations as well as experimental procedures. There were differences in the inhibitory potency of the same compounds isolated from different seaweed species against α-glucosidase activity. As discussed above, dieckol is one example. BDDE (IC_50_ = 0.03 μM) from *S. latiuscula* [135] has more inhibitory activity compared with BDDE (IC_50_ = 0.098 μM) derived from *O. corymbifera* [135] and *P. lancifolia* [137]. Similarly, eckol (IC_50_ = 11.16 μM) from *E. maxima* [141] was more potent compared with the same compound (IC_50_ = 22.78 μM) isolated from *E. stolonifera* [138]. Other notable examples included dioxinodehydroeckol [134,138] and 7-phloroeckol [138,139]. Indeed, there were seasonal and species-related differences in the carbolytic enzyme inhibitory activities by fucoidan isolated from *Ascophyllum nodosum* and *Fucus vesiculosus* [232]. Fucoidan, water-soluble polysaccharide inhibited α-glucosidase and α-amylase activities differently depending on the collection period and target enzyme. Fucoidan extracted from *A. nodosum* inhibited both α-glucosidase and α-amylase whereas fucoidan from *F. vesiculosus* is only active against α-glucosidase. Fucoidan from *A. nodosum* also reduced α-amylase activity between 7% and 100% at 5 mg/mL with IC_50_ values of 0.12 to 4.64 mg/mL depending on the seaweed collection period. This inhibitory difference was primarily due to chemical structure and molecular weight of the fucoidans extracted from these two species. Therefore, seaweed bioactive compounds are susceptible to the influence of environment and other various factors. Seasonal and within-plant variations of seaweed contents have been reported such as in phenolics against α-glucosidase in *Ascophyllum nodosum* [233], as well as fatty acid content and composition in *Spatoglossum macrodontum* [234]. Furthermore, extraction techniques and other experimental methods might also influence the types of compounds isolated. This could possibly explain the differences in various compounds isolated from the same species of seaweed and also potency differences of the same compounds from different species of seaweed. On the other hand, several anti-diabetic compounds isolated from seaweed were found to possess possibly more than one activity. This also reflected the fact that different compounds received more attention than others in the literature. Dieckol was reported to exhibit various anti-diabetic activities including inhibition against α-glucosidase, α-amylase, lipase, aldose reductase, PTP1B, and AGE-formation. Dieckol is also anti-oxidative and capable of increasing hepatic antioxidant enzymes activities. Similarly, eckol, dioxinodehydroeckol, 7-phloroeckol and fucoxanthin have also been identified to possess anti-diabetic activities via different routes.

## 4. Conclusions and Perspectives

In this review, we have discussed the contents and diverse bioactive compounds isolated from seaweed, which have been demonstrated to be potentially beneficial in T2DM management. Indeed, seaweeds especially the edible species contain numerous compounds with different modes of action involving both specific mechanisms such as interaction with crucial proteins and broad non-specific mechanisms including anti-inflammatory response and up-regulation of antioxidant enzymes. All these can be suitably employed in T2DM treatment strategies. Polysaccharides and dietary fibres from seaweed may improve post-prandial satiety feeling, thereby they assist in reduction of blood glucose levels and improved insulin sensitivity. Seaweed dietary fibres are also helpful in reducing body weight or weight maintenance, hence they are beneficial in attenuating the risk of obesity. Dietary fibres, MUFA and PUFA are also beneficial in mitigating inflammatory response due to hyperglycaemia and adiposity. Phenolic and non-phenolic compounds isolated from seaweed have positive impacts on the recognised hallmarks of T2DM such as hyperglycaemia and hyperlipidaemia. Generally, these seaweed bioactive compounds’ modes of action are comparably similar to other known anti-diabetic drugs. Their ability to inhibit carbohydrate carbolytic enzymes such as α-amylase and α-glucosidase, hence, reducing the rate of carbohydrate digestion and absorption is similar to acarbose whereas improvement of insulin sensitivity and incretin hormones stimulation are similar to metformin and DPP-4 inhibitors respectively. Furthermore, extracts and bioactive compounds from seaweed may also exert their anti-diabetic effects by increasing cellular uptake of glucose, inhibition of enzymes such as aldose reductase, PTP1B and DPP-4, attenuation of AGE formation, β-cell cytoprotection as well as action against obesity and the associated inflammatory responses. Therefore, seaweed and seaweed-derived bioactive compounds possess huge potential to be employed in T2DM management either as part of dietary intake or as purified pharmacological agents and supplements. Considering that T2DM takes time to develop, dietary intake of seaweed for glycaemic control as preventive measures should be explored. Further research in the future is required to fully capitalise on the benefits of seaweed bioactive compounds in preventing and managing T2DM.

## Abbreviations

Adrb3: β3-adrenergic receptor
AGE: advanced glycation end-products
AMPK: 5′adenosine monophosphate-activated protein kinase
ALT: alanine transaminase
ARE: antioxidant-response element
AST: aspartate transaminase
ATP: adenosine triphosphate
BDDE: bis (2,3-dibromo-4,5-dihydroxybenzyl) ether
BIP: butyl-isobutylphthalate
CAT: catalase
COX-2: cyclooxygenase-2
DHA: docosahexaenoic acid
DDBT: 2-(4-(3,5-dihydroxyphenoxy)-3,5-dihydroxyphenoxy) benzene-1,3,5-triol
DPHC: diphlorethohydroxycarmalol
DPP-4: dipeptidyl-peptidase-4
DPPH: 2,2-diphenyl-1-picrylhydrazyl
GAE: gallic acid equivalents
GIP: glucose-dependent insulinotrophic polypeptide
GLP-1: glucagon-like peptide-1
GLUT1: glucose transporter type-1
GLUT4: glucose transporter type-4
GSH-px: glutathione peroxidase
HbA1C: glycosylated haemoglobin
IL-6: interleukin-6
IL-10: interleukin-10
IL-1β: interleukin -1β
IRS-1: insulin receptor substrate-1
IRS-1/PI3K: insulin receptor substrate-1/phosphoinositide 3-kinase
iNOS: inducible nitric oxide synthase
MUFA: monounsaturated fatty acids
MCP-1: monocyte chemoattractant protein-1
mRNA: messenger ribonucleic acid
MnSOD: manganese superoxide dismutase
NF-κB: nuclear factor-kappaB
PAI-1: plasminogen activator inhibitor-1
PEG-2: prostaglandin E2
PI3K/Akt: phosphoinositide 3-kinase/Akt
PPAR-γ: peroxisome proliferator-activated receptor-γ
PTP1B: protein tyrosine phosphatase 1B
PUFA: polyunsaturated fatty acids
ROS: reactive oxygen species
SOD: superoxide dismutase
T1DM: Type I Diabetes Mellitus
T2DM: Type II Diabetes Mellitus
TBARS: thiobarbituric acid reactive substances
TLR: toll-like receptors
TNF-α: tumour necrosis factor-α
UCP-1: uncoupling protein-1
WHO: World Health Organisation

## References

[1] Cefalu W.T. (2007). Pharmacotherapy for the treatment of patients with type 2 diabetes mellitus: Rationale and specific agents. Clin. Pharmacol. Ther..

[2] Wild S., Roglic G., Green A., Sicree R., King H. (2004). Global prevalence of diabetes. Estimates for the year 2000 and projections for 2030. Diabetes Care.

[3] American Diabetes Association (2008). Standards for medical care in diabetes. Diabetes Care.

[4] Beckman J.A., Creager M.A., Libby P. (2002). Diabetes and atherosclerosis: Epidemiology, pathophysiology and management. JAMA.

[5] Booth G.L., Kapral M.K., Fung K., Tu J.V. (2006). Relation between age and cardiovascular disease in men and women with diabetes compared with non-diabetic people: A population-based retrospective cohort study. Lancet.

[6] Roglic G., Unwin N., Bennett P.H., Mathers C., Tuomilehto J., Nag S., Connolly V., King H. (2005). The burden of mortality attributable to diabetes. Realistic estimates for the year 2000. Diabetes Care.

[7] Roglic G., Unwin N. (2009). Mortality attributable to diabetes: Estimates for the year 2010. Diabetes Res. Clin. Pract..

[8] Zimmet P. (1999). Diabetes epidemiology as a trigger to diabetes research. Diabetologia.

[9] Scheen A.J., Lefebvre P.J. (1996). Insulin action in man. Diabetes Metab..

[10] Aronoff S.L. (2004). Glucose metabolism and regulation: Beyond insulin and glucagon. Diabetes Spectr..

[11] Van Haeften T.W., Pimenta W., Mitrakou A., Korytkowski M., Jenssen T., Yki-Jarvinen H., Gerich J.E. (2000). Relative contributions of beta-cell function and tissue insulin sensitivity to fasting and postglucose-load glycemia. Metabolism.

[12] Mitrakou A., Kelley D., Mokan M., Veneman T., Pangburn T., Reilly J., Gerich J. (1992). Role of reduced suppression of glucose production and diminished early insulin release in impaired glucose tolerance. N. Engl. J. Med..

[13] Meyer C., Woerle H.J., Dostou J.M., Welle S.L., Gerich J.E. (2004). Abnormal renal, hepatic and muscle glucose metabolism following glucose ingestion in type 2 diabetes. Am. J. Physiol. Endocrinol. Metab..

[14] Woerle H.J., Szoke E., Meyer C., Dostou J.M., Wittlin S.D., Gosmanov N.R., Welle S.L., Gerich J.E. (2006). Mechanisms for abnormal post-prandial glucose metabolism in type 2 diabetes. Am. J. Physiol. Endocrinol. Metab..

[15] Diabetes Control and Complications Trial Research Group (1993). The effect of intensive treatment of diabetes on the development and progression of long-term complications in insulin-dependent diabetes mellitus. N. Engl. J. Med..

[16] United Kingdom Prospective Diabetes Study Group (UKPDS) (1998). Intensive blood-glucose control with sulfonylureas or insulin compared with conventional treatment and risk of complications in patients with Type 2 diabetes (UKPDS 33). Lancet.

[17] Pan X.R., Li G.W., Hu Y.H., Wang J.X., Yang W.Y., An Z.X., Hu Z.X., Lin J., Xiao J.Z., Cao H.B. (1997). Effects of diet and exercise in preventing NIDDM in people with impaired glucose tolerance. The Da Qing IGT and diabetes study. Diabetes Care.

[18] Tuomilehto J., Lindström J., Eriksson J.G., Valle T.T., Hämäläinen H., Ilanne-Parikka P., Keinänen-Kiukaanniemi S., Laakso M., Louheranta A., Rastas M. (2001). Prevention of type 2 diabetes mellitus by changes in lifestyle among subjects with impaired glucose tolerance. N. Engl. J. Med..

[19] Misra A. (2009). Prevention of Type 2 diabetes: The long and winding road. Lancet.

[20] Diabetes Prevention Program Research Group (2009). 10-year follow-up of diabetes incidence and weight loss in the Diabetes Prevention Program Outcome Study. Lancet.

[21] Holman R.R., Paul S.K., Bethel M.A., Matthews D.R., Neil H.A. (2008). 10-year follow-up of intensive glucose control in type 2 diabetes. N. Engl. J. Med..

[22] Van Dam R.M., Rimm E.B., Willett W.C., Stampfer M.J., Hu F.B. (2002). Dietary patterns and risk for type 2 diabetes mellitus in U.S. men. Ann. Intern. Med..

[23] Montonen J., Knekt P., Harkanen T., Jarvinen R., Heliovaara M., Aromaa A., Reunanen A. (2005). Dietary patterns and the incidence of type 2 diabetes. Am. J. Epidemiol..

[24] Jiménez-Escrig A., Sáchez-Muniz F.J. (2000). Dietary fibre from edible seaweeds: Chemical structure, physicochemical properties and effects on cholesterol metabolism. Nutr. Res..

[25] Yang Y.J., Nam S.J., Kong G., Kim M.K. (2010). A case-control study on seaweed consumption and the risk of breast cancer. Br. J. Nutr..

[26] Matsumura Y. (2001). Nutrition trends in Japan. Asia Pac. J. Clin. Nutr..

[27] Kadam S.U., Prabhasankar P. (2010). Marine foods as functional ingredients in bakery and pasta products. Food Res. Int..

[28] Carvalho A.F.U., Portela M.C.C., Sousa M.B., Martins F.S., Rocha F.C., Farias D.F., Feitosa J.P.A. (2009). Physiological and physico-chemical characterization of dietary fibre from the green seaweed *Ulva fasciata* Delile. Braz. J. Biol..

[29] Cofrades S., López- López I., Bravo L., Ruiz-Capillas C., Bastida S., Larrea M.T., Jiménez-Colmenero F. (2010). Nutritional and antioxidant properties of different brown and red Spanish edible seaweeds. Food Sci. Technol. Int..

[30] Siró I., Kápolna E., Kápolna B., Lugasi A. (2008). Functional food. Product development, marketing and consumer acceptance- a review. Appetite.

[31] Honkanen P., Luten J. (2009). Consumer Acceptance of (Marine) Functional Food. Marine Functional Food.

[32] Iso H. (2011). Lifestyle and cardiovascular disease in Japan. Atheroscler. Thromb..

[33] Kim J., Shin A., Lee J.S., Youn S., Yoo K.Y. (2009). Dietary factors and breast cancer in Korea: An ecological study. Breast J..

[34] Drewnowski A., Popkin B.M. (1997). The nutrition transition: New trends in the global diet. Nutr. Rev..

[35] Iso H., Date C., Noda H., Yoshimura T., Tamakoshi A., JACC Study Group (2005). Frequency of food intake and estimated nutrient intake among men and women: The JACC Study. J. Epidemiol..

[36] Nagataki S. (2008). The average of dietary iodine intake due to the ingestion of seaweed is 1.2 mg/day in Japan. Thyroid.

[37] Rupérez P. (2002). Mineral content of edible marine seaweeds. Food Chem..

[38] MacArtain P., Gill C.I.R., Brooks M., Campbell R., Rowland I.R. (2007). Nutritional value of edible seaweeds. Nutr. Rev..

[39] Bocanegra A., Bastida S., Benedi J., Ródenas S., Sánchez-Muniz F.J. (2009). Characteristics and nutritional and cardiovascular-health properties of seaweeds. J. Med. Food.

[40] Burtin P. (2003). Nutritional value of seaweeds. EJEAFChe.

[41] Jurkovic N., Kolb N., Colic I. (1995). Nutritive value of marine algae *Laminaria japonica* and *Undaria pinnatifida*. Nahrung.

[42] Urbano M.G., Goni I. (2002). Bioavailability of nutrients in rats fed on edible seaweeds, nori (*Porphyra tenera*) and wakame (*Undaria pinnatifida*) as a source of dietary fibre. Food Chem..

[43] Lee H.Y., Won J.C., Kang Y.J. (2010). Type 2 diabetes in urban and rural districts in Korea: Factors associated with prevalence difference. J. Korean Med. Sci..

[44] Lee H.J., Kim H.C., Vitek L., Nam C.M. (2010). Algae consumption and risk of type 2 diabetes: Korean national health and nutrition examination survey in 2005. J. Nutr. Sci. Vitaminol..

[45] Paradis M.E., Couture P., Lamarche B. (2011). A randomised crossover placebo-controlled trial investigating the effect of brown seaweed (*Ascophyllum nodosum* and *Fucus vesiculosus*) on post-challenge plasma glucose and insulin levels in men and women. Appl. Physiol. Nutr. Metab..

[46] Tanemura Y., Yamanaka-Okumura H., Sakuma M., Nii Y., Taketani Y., Takeda E. (2014). Effects of the intake of *Undaria pinnatifida* (Wakame) and its sporophylls (Mekabu) on postprandial glucose and insulin metabolism. J. Med. Investig..

[47] Yeon J.Y., Bae Y.J., Kim E.Y., Lee E.J. (2015). Association between flavonoid intake and diabetes risk among Koreans. Clin. Chim. Acta.

[48] Vessby B., Gustafsson I.B., Boberg J., Karlstrom B., Lithell H., Werner I. (1980). Substituting polyunsaturated for saturated fat as a single change in a Swedish diet: Effects on serum lipoprotein metabolism and glucose tolerance in patients with hyperlipoproteinaemia. Eur. J. Clin. Investig..

[49] Vessby B., Uusitupa M., Hermansen K., Riccardi G., Rivellese A.A., Tapsell L.C., Nälsén C., Berglund L., Louheranta A., Rasmussen B.M. (2001). Substituting dietary saturated for monounsaturated fat impairs insulin sensitivity in healthy men and women: The KANWU Study. Diabetologia.

[50] Borkman M., Campbell L.V., Chisholm D.J., Storlien L.H. (1991). Comparison of the effects on insulin sensitivity of high carbohydrate and high fat diets in normal subject. J. Clin. Endocrinol. Metab..

[51] Garg A., Grundy S.M., Unger R.H. (1992). Comparison of effects of high and low carbohydrate diets on plasma lipoproteins and insulin sensitivity in patients with mild NIDDM. Diabetes.

[52] Riserus U., Willett W.C., Hu F.B. (2009). Dietary fats and prevention of type 2 diabetes. Prog. Lipid Res..

[53] Dimopoulos N., Watson M., Sakamoto K., Hundal H.S. (2006). Differential effects of palmitate and palmitoleate on insulin action and glucose utilization in rat L6 skeletal muscle cells. Biochem. J..

[54] Sabin M.A., Moon J.H., Lee J.Y., Kang S.B., Park J.S., Lee B.W., Kang E.S., Ahn C.W., Lee H.C., Cha B.S. (2010). Dietary monounsaturated fatty acids preserve the insulin signaling pathway via IRS-1/PI3K in rat skeletal muscle. Lipids.

[55] Thomsen C., Storm H., Holst J.J., Hermansen K. (2003). Differential effects of saturated and monounsaturated fats on postprandial lipemia and glucagon-like peptide 1 responses in patients with type 2 diabetes. Am. J. Clin. Nutr..

[56] Esposito K., Maiorino M.I., Ciotola M., di Palo C., Scognamiglio P., Gicchino M., Petrizzo M., Saccomanno F., Beneduce F., Ceriello A. (2009). Effects of a Mediterranean-style diet on the need for anti-hyperglycemic drug therapy in partients with newly diagnosed type 2 diabetes: A randomized trial. Ann. Intern. Med..

[57] Spranger J., Kroke A., Möhlig M., Bergmann M.M., Ristow M., Boeing H., Pfeiffer A.F. (2003). Adiponectin and protection against type 2 diabetes mellitus. Lancet.

[58] Wang R., Paul V.J., Luesch H. (2013). Seaweed extracts and unsaturated fatty acid constituents from the green alga *Ulva lactuca* as activators of the cytoprotective Nrf2-ARE pathway. Free Radic. Biol. Med..

[59] Gill I., Valivety R. (1997). Polyunsaturated fatty acids: Part 1. Ocurrence, biological activities and application. Trends Biotechnol..

[60] Funk C.D. (2001). Prostaglandins and leukotrienes: Advances in eicosanoids biology. Science.

[61] Napier J.A., Michaelson L.V., Stobart A.K. (1999). Plant desaturases: Harvesting the fat of the land. Curr. Opin. Plant Biol..

[62] Sanchez-Machado D.I., Lopez-Hernandez J., Paseiro-Losada P., Lopez-Cervantes J. (2004). Fatty acids, total lipid, protein and ash contents of processed edible seaweeds. Food Chem..

[63] De Angelis L., Rise P., Giavarini F., Bolis C.L., Colombo M.L. (2005). Marine macroalgae analyzed by mass spectrometry are rich sources of polyunsaturated fatty acids. J. Mass Spectrom..

[64] Colombo M.L., Rise P., Giavarini F., de Angelis L., Galli C., Bolis C.L. (2006). Marine macroalgae as sources of polyunsaturated fatty acids. Plant Foods Hum. Nutr..

[65] Pereira H., Barreira L., Figueiredo F., Custodio L., Vizetto-Duarte C., Polo C., Resek E., Engelen A., Varel J. (2012). Polyunsaturated fatty acids of marine macroalgae: Potential for nutritional and pharmaceutical applications. Mar. Drugs.

[66] Marsham S., Scott G.W., Tobin M.L. (2007). Comparison of nutritive chemistry of a range of temperate seaweeds. Food Chem..

[67] Lunn J., Theobald H.E. (2006). The health effects of dietary unsaturated fatty acids. Br. Nutr. Found. Nutr. Bull..

[68] Kumari P., Kumar M., Gupta V., Reddy C.R.K., Jha B. (2010). Tropical marine macroalgae as potential sources of nutritionally important PUFAs. Food Chem..

[69] Summers L.K., Fielding B.A., Bradshaw H.A., Ilic V., Beysen C., Clark M.L., Moore N.R., Frayn K.N. (2002). Substituting dietary saturated fat with polyunsaturated fat changes abdominal fat distribution and improves insulin sensitivity. Diabetologia.

[70] Feskens E.J. (2001). Can diabetes be prevented by vegetable fat?. Diabetes Care.

[71] Hu F.B., van Dam R.M., Liu S. (2001). Diet and risk of type II diabetes: The role of types of fat and carbohydrate. Diabetologia.

[72] Marshall J.A., Bessesen D.H. (2002). Dietary fat and the development of type 2 diabetes. Diabetes Care.

[73] Kamada T., Yamashita T., Baba Y., Kai M., Setoyama S., Chuman Y., Otsuji S. (1986). Dietary sardine oil increases erythrocyte membrane fluidity in diabetic patients. Diabetes.

[74] Vessby B. (2000). Dietary fat and insulin action in humans. Br. J. Nutr..

[75] Lovejoy J.C. (2002). The influence of dietary fat on insulin resistance. Curr. Diabetes Rep..

[76] Fedor D., Kelley D.S. (2009). Prevention of insulin resistance by n-3 polyunsaturated fatty acids. Curr. Opin. Nutr. Metab. Care.

[77] Lee J.Y., Zhao L., Youn H.S., Weatherill A.R., Tapping R., Feng L., Lee W.H., Fitzgerald K.A., Hwang D.H. (2004). Saturated fatty acid activates but polyunsaturated fatty acid inhibits toll-like receptor 2 dimerized with toll-like receptor 6 or 1. J. Biol. Chem..

[78] Ferrucci L., Cherubini A., Bandinelli S., Bartali B., Corsi A., Lauretani F., Martin A., Andres-Lacueva C., Senin U., Guralnik J.M. (2006). Relationship of plasma polyunsaturated fatty acids to circulating inflammatory markers. J. Clin. Endocrinol. Metab..

[79] Hartweg J., Farmer A.J., Perera R., Holman R.R., Neil H.A. (2007). Meta-analysis of the effects of n-3 polyunsaturated fatty acids on lipoproteins and other emerging lipid cardiovascular risk markers in patients with type 2 diabetes. Diabetologia.

[80] Khan N.A., Choi J.S., Lee M.C., Kim E., Nam T.J., Fujii H., Hong Y.K. (2008). Anti-inflammatory activities of methanol extracts from various seaweed species. J. Environ. Biol..

[81] Jump D.B., Botolin D., Wang Y., Xu J., Christian B., Demeure O. (2005). Fatty acid regulation of hepatic gene transcription. J. Nutr..

[82] De Munter J.S., Hu F.B., Spiegelman D., Franz M., van Dam R.M. (2007). Whole grain, bran, and germ intake and risk of type 2 diabetes: A prospective cohort study and systematic review. PLoS Med..

[83] Schulze M.B., Schulz M., Heidemann C., Schienkiewitz A., Hoffmann K., Boeing H. (2007). Fiber and magnesium intake and incidence of type 2 diabetes: A prospective study and meta-analysis. Arch. Intern. Med..

[84] Mann J.I., de Leeuw I., Hermansen K., Karamanos B., Karlström B., Katsilambros N., Riccardi G., Rivellese A.A., Rizkalla S., Slama G. (2004). Evidence-based nutritional approaches to the treatment and prevention of diabetes mellitus. Nutr. Metab. Cardiovasc. Dis..

[85] American Diabetes Association (2005). Position statement: Standards of medical care in diabetes. Diabetes Care.

[86] Kim M.S., Kim J.Y., Choi W.H., Lee S.S. (2008). Effects of seaweed supplementation on blood glucose concentration, lipid profile and anti-oxidant enzyme activities in patients with type 2 diabetes mellitus. Nutr. Res. Pract..

[87] Ludwig D.S., Pereira M.A., Kroenke C.H., Hilner J.E., van Horn L., Slattery M.L., Jacobs D.R. (1999). Dietary fiber, weight gain and cardiovascular disease risk factors in young adults. JAMA.

[88] Howarth N.C., Saltzman E., Roberts S.B. (2001). Dietary fiber and weight regulation. Nutr. Rev..

[89] Weickert M.O., Pfeiffer A.F. (2008). Metabolic effects of dietary fibre consumption and prevention of diabetes. J. Nutr..

[90] Toeller M., Buyken A.E., Heitkamp G., de Pergola G., Giorgino F., Fuller J.H. (1999). Fiber intake, serum cholesterol levels and cardiovascular disease in European individuals with type 1 diabetes. EURODIAB IDDM Complications Study Group. Diabetes Care.

[91] King D.E., Egan B.M., Woolson R.F., Mainous A.G., Al-Solaiman Y., Jesri A. (2007). Effect of a high fiber diet *vs.* a fiber-supplemented diet on C-reactive protein level. Arch. Intern. Med..

[92] Mellen P.B., Liese A.D., Tooze J.A., Vitolins M.Z., Wagenknecht L.E., Herrington D.M. (2007). Whole grain intake and carotid artery atherosclerosis in a multiethnic cohort: The Insulin Resistance Atherosclerosis Study. Am. J. Clin. Nutr..

[93] Pi-Sunyer X. (2005). Do glycemic index, glycemic load and fiber play a role in insulin sensitivity, disposition index, and type 2 diabetes?. Diabetes Care.

[94] Dumelod B.D., Ramirez R.P., Tiangson C.L., Barrios E.B., Panlasiqui L.N. (1999). Carbohydrate availability of arroz caldo with lambda-carrageenan. Int. J. Food Sci. Nutr..

[95] Goni I., Valdivieso L., Garcia-Alonso A. (2000). Nori seaweed consumption modifies glycemic response in healthy volunteers. Nutr. Res..

[96] Lamela M., Anca J., Villar R., Otero J., Calleja J.M. (1989). Hypoglycemic activity of several seaweed extracts. J. Ethnopharmacol..

[97] Lamela M., Anca J.M., Vazquez-Freire M.J., Gato A., Calleja J.M. (1996). Hypoglycemic Activity of a fucan from Himanthalia elongata. Médicaments et Aliments, Approche Ethnopharmacologique: Actes du 2e Colloque Européen D’ethnopharmacologie et de la 11e Conférence Internationale D’ethnomédecine.

[98] Vaugelade P., Hoebler C., Francoise B., Guillon F., Lahaye M., Duee P., Darcy-Vrillon B. (2000). Non-starch polysaccharides extracted from seaweed can modulate intestinal absorption of glucose and insulin response in the pig. Reprod. Nutr. Dev..

[99] Zhang D., Fujii I., Lin C., Ito K., Guan H., Zhao J., Shinohara M., Matsukura M. (2008). The stimulatory activities of polysaccharide compounds derived from algae extracts on insulin secretion *in vitro*. Biol. Pharm. Bull..

[100] Pereira M.A., Ludwig D.S. (2001). Dietary fiber and body-weight regulation. Observations and mechanisms. Pediatr. Clin. North Am..

[101] Howarth N.C., Saltzman E., McCrory M.A., Greenberg A.S., Dwyer J., Ausman L., Kramer D.G., Roberts S.B. (2003). Fermentable and non-fermentable fiber supplements did not alter hunger, satiety or body weight in a pilot study of men and women consuming self-selected diets. J. Nutr..

[102] Weickert M.O., Spranger J., Holst J.J., Otto B., Koebnick C., Mohlig M., Pfeiffer A.F. (2006). Wheat-fibre-induced changes of post-prandial peptide YY and ghrelin responses are not associated with acute alterations of satiety. Br. J. Nutr..

[103] Heini A.F., Lara-Castro C., Schneider H., Kirk K.A., Consinidine R.V., Weinser R.L. (1998). Effect of hydrolyzed guar fiber on fasting and postprandial satiety and satiety hormones: A double-blind, placebo-controlled trial during controlled weight loss. Int. J. Obes. Relat. Metab. Discord..

[104] Robertson M.D., Currie J.M., Morgan L.M., Jewell D.P., Frayn K.N. (2003). Prior short-term consumption of resistant starch enhances postprandial insulin sensitivity in healthy subjects. Diabetologia.

[105] Weickert M.O., Mohlig M., Koebnick C., Holst J.J., Namsolleck P., Ristow M., Osterhoff M., Rochlitz H., Rudovich N., Spranger J. (2005). Impact of cereal fibre on glucose-regulating factors. Diabetologia.

[106] Weickert M.O., Pfeiffer A.F. (2006). Signalling mechanisms linking hepatic glucose and lipid metabolism. Diabetologia.

[107] Kristensen M., Jensen M.G. (2011). Dietary fibres in the regulation of appetite and food intake. Importance of viscosity. Appetite.

[108] Hoad C.L., Rayment P., Spiller R.C., Marciani L., Alonso Bde C., Traynor C., Mela D.J., Peters H.P., Gowland P.A. (2004). *In vivo* imaging of intragastric gelation and its effects on satiety in humans. J. Nutr..

[109] Mattes R.D. (2007). Effects of a combination fiber system on appetite and energy intake in overweight humans. Physiol. Behav..

[110] Pelkman C.L., Navia J.L., Miller A.E., Pohle R.J. (2007). Novel calcium-gelled, alginate-pectin beverage reduced energy intake in non-dieting overweight and obese women: Interactions with dietary restraint status. Am. J. Clin. Nutr..

[111] Paxman J.R., Richardson J.C., Dettmar P.W., Corfe B.M. (2008). Daily ingestion of alginate reduces energy intake in free-living subjects. Appetite.

[112] Paxman J.R., Richardson J.C., Dettmar P.W., Corfe B.M. (2008). Alginate reduces the increased uptake of cholesterol and glucose in overweight male subjects: A pilot study. Nutr. Res..

[113] Wolf B.W., Lai C.S., Kipnes M.S., Ataya D.G., Wheeler K.B., Zinker B.A., Garleb K.A., Firkins J.L. (2002). Glycemic and insulinemic responses of nondiabetic healthy adult subjects to an experimental acid-induced viscosity complex incorporated into a glucose beverage. Nutrition.

[114] Williams J.A., Lai C.S., Corwin H., Ma Y., Maki K.C., Garleb K.A., Wolf B.W. (2004). Inclusion of guar gum and alginate into crispy bar improves post-prandial glycemia in humans. J. Nutr..

[115] An C., Kuda T., Yazaki T., Takahashi H., Kimura B. (2013). FLX pyrosequencing analysis of the effects of the brown-algal fermentable polysaccharides alginate and laminaran on rat cecal microbiotas. Appl. Environ. Microbiol..

[116] Wolever T.M., Spadafora P.J., Cunnane S.C., Pencharz P.B. (1995). Propionate inhibits incorporation of colonic [1,2-^13^C]acetate into plasma lipids in humans. Am. J. Clin. Nutr..

[117] Odunsi S.T., Vazquez-Roque M.I., Camilleri M., Papathanasopoulos A., Clark M.M., Wodrich L., Lempke M., McKinzie S., Ryks M., Burton D. (2010). Effect of alginate on satiation, appetite, gastric function and selected gut satiety hormones in overweight and obesity. Obesity.

[118] Georg Jensen M., Kristensen M., Belza A., Knudsen J.C., Astrup A. (2012). Acute effect of alginate-based preload on satiety feelings, energy intake, and gastric emptying rate in healthy subjects. Obesity.

[119] Sattarahmady N., Khodagholi F., Moosavi-Mohavedi A.A., Heli H., Hakimelahi G.H. (2007). Alginate as an antiglycating agent for human serum albumin. Int. J. Biol. Macromol..

[120] Hall A.C., Fairclough A.C., Mahadevan K., Paxman J.R. (2012). *Ascophyllum nodosum* enriched bread reduces subsequent energy intake with no effect on post-prandial glucose and cholesterol in healthy, overweight males. A pilot study. Appetite.

[121] Galisteo M., Duarte J., Zarzuelo A. (2008). Effects of dietary fibers on disturbances clustered in the metabolic syndrome. J. Nutr. Biochem..

[122] Vos A.P., M’Rabet L., Stahl B., Boehm G., Garssen J. (2007). Immune-modulatory effects and potential working mechanisms of orally applied non-digestable carbohydrates. Crit. Rev. Immunol..

[123] Qi L., Hu F.B. (2007). Dietary glycemic load, whole grains and systemic inflammation in diabetes: The epidemiological evidence. Curr. Opin. Lipidol..

[124] Kim K.J., Lee B.Y. (2012). Fucoidan from the sporophyll of *Undaria pinnatifida* suppresses adipocyte differentiation by inhibition of inflammation-related cytokines in 3T3-L1 cells. Nutr. Res..

[125] Lumeng C.N. (2013). Innate immune activation in obesity. Mol. Asp. Med..

[126] Taboada C., Millan R., Miguez I. (2010). Composition, nutritional aspects and effect on serum parameters of marine alga *Ulva rigida*. J. Sci. Food Agric..

[127] BelHadj S., Hentati O., Abdelfattah E., Hamden K. (2013). Inhibitory activities of *Ulva lactuca* polysaccharides on digestive enzymes related to diabetes and obesity. Arch. Physiol. Biochem..

[128] Stern J.L., Hagerman A.E., Steinberg P.D., Mason P.K. (1996). Phlorotannins-protein interactions. J. Chem. Ecol..

[129] Anhe F.F., Desjardins Y., Pilon G., Dudonne S., Genovese M.I., Lajolo F.M., Marette A. (2013). Polyphenols and type 2 diabetes: A prospective review. PharmaNutrition.

[130] Casirola D.M., Ferraris R.P. (2006). α-Glucosidase inhibitors prevent diet-induced increases in intestinal sugar transport in diabetic mice. Metabolism.

[131] Teixera V.L., Rocha F.D., Houghton P.J., Kaplan M.A., Pereira R.C. (2007). α-Amylase inhibitors from Brazillian seaweeds and their hypoglycaemic potential. Fitoterapia.

[132] Chin Y.X., Lim P.E., Maggs C.A., Phang S.M., Sharifuddin Y., Green B.D. (2014). Anti-diabetic potential of selected Malaysian seaweeds. J. Appl. Phycol..

[133] Okada Y., Ishimaru A., Suzuki R., Okuyama T. (2004). A new phloroglucinol derivative from the brown alga *Eisenia bicyclis*: Potential for the effective treatment of diabetic complications. J. Nat. Prod..

[134] Eom S.H., Lee S.H., Yoon N.Y., Jung W.K., Jeon Y.J., Kim S.K., Lee M.S., Kim Y.M. (2012). α-Glucosidase and α-amylase-inhibitory activities of phlorotannins from *Eisenia bicyclis*. J. Sci. Food Agric..

[135] Kurihara H., Mitani T., Kawabata J., Takahashi K. (1999). Inhibitory potencies of bromophenols from Rhodomelaceae algae against α-glucosidase activity. Fish. Sci..

[136] Iwai K. (2008). Antidiabetic and antioxidant effects of polyphenols in brown alga *Ecklonia stolonifera* in genetically diabetic KK-A^y^ mice. Plant Foods Hum. Nutr..

[137] Kim K.Y., Nguyen T.H., Kurihara H., Kim S.M. (2010). α-Glucosidase inhibitory activity of bromophenol purified from the red alga *Polyopes lancifolia*. J. Food Sci..

[138] Moon H.E., Islam N., Ahr B.R., Chowdhury S.S., Sohn H.S., Jung H.A., Choi J.S. (2011). Protein tyrosine phosphatase 1B and α-glucosidase inhibitory phlorotannins from edible brown algae *Ecklonia stolonifera* and *Eisenia bicyclis*. Biosci. Biotechnol. Biochem..

[139] Lee S.H., Li Y., Karadeniz F., Kim M.M., Kim S.K. (2009). α-Glucosidase and α-amylase inhibitory activities of phloroglucinol derivatives from edible marine brown alga, *Ecklonia cava*. J. Sci. Food Agric..

[140] Lee S.H., Park M.H., Heo S.J., Kang S.M., Ko S.C., Han S.J., Jeon Y.J. (2010). Dieckol isolated from *Ecklonia cava* inhibits α-glucosidase and α-amylase *in vitro* and alleviates post-prandial hyperglycemia in streptozotocin-induced diabetic mice. Food Chem. Toxicol..

[141] Rengasamy K.R., Aderogba M.A., Amoo S.O., Stirk W.A., van Staden J. (2013). Potential antiradical and α-glucosidase inhibitors from *Ecklonia maxima* (Osbeck) Papenfuss. Food Chem..

[142] Kim K.Y., Nam K.A., Kurihara H., Kim S.M. (2008). Potent α-glucosidase inhibitors purified from the red algae *Grateloupia elliptica*. Phytochemistry.

[143] Heo S.J., Hwang J.Y., Choi J.I., Han J.S., Kim H.J., Jeon J.Y. (2009). Diphlorethohydroxycarmalol isolated from *Ishige okamurae*, a brown algae, a potent α-glucosidase and α-amylase inhibitor, alleviates postprandial hyperglycaemia in diabetic mice. Eur. J. Pharmacol..

[144] Bu T., Liu M., Zheng L., Guo Y., Lin X. (2010). α-Glucosidase inhibition and the *in vivo* hypoglycaemic effect of butyl-isobutyl-phthalate derived from the *Laminaria japonica* rhizoid. Phytother. Res..

[145] Zhang J., Tiller C., Shen J., Wang C., Girouard G.S., Dennis D., Barrow C.J., Miao M., Ewart H.S. (2007). Antidiabetic properties of polysaccharide- and polyphenolic-enriched fractions from the brown seaweed *Ascophyllum nodosum*. Can. J. Physiol. Pharmacol..

[146] Apostolidis E., Lee C.M. (2010). *In vitro* potential of *Ascophyllum nodosum* phenolic antioxidant mediated α-glucosidase and α-amylase inhibition. J. Food Sci..

[147] Nwosu F., Morris J., Lund V.A., Stewart D., Ross H.A., McDougall G.J. (2011). Anti-proliferative and potential anti-diabetic effects of phenolic-rich extracts from marine edible algae. Food Chem..

[148] Lordan S., Smyth T.J., Soler-Vila A., Stanton C., Ross R.P. (2013). The α-amylase and α-glucosidase inhibitory effects of Irish seaweed extracts. Food Chem..

[149] Kellogg J., Grace M.H., Lila M.A. (2014). Phlorotannins from Alaskan seaweed inhibit carbolytic enzyme activity. Mar. Drugs.

[150] Park M.H., Han S.J. (2012). Hypoglycemic effect of *Padina arborescens* extract in streptozotocin-induced diabetic mice. Prev. Nutr. Food Sci..

[151] Kawamura-Konishi Y., Watanabe N., Saito M., Nakajima N., Sasaki T., Katayama T., Enomoto T. (2012). Isolation of a new phlorotannin, a potent inhibitor of carbohydrate-hydrolyzing enzymes, from the brown alga *Sargassum patens*. J. Agric. Food Chem..

[152] Lee C.W., Han J.S. (2012). Hypoglycemic effect of *Sargassum ringgoldianum* extract in STZ-induced diabetic mice. Prev. Nutr. Food Sci..

[153] Hwang P.A., Hung Y.L., Tsai Y.K., Chien S.Y., Kong Z.L. (2015). The brown seaweed *Sargassum hemiphyllum* exhibits α-amylase and α-glucosidase inhibitory activity and enhances insulin release *in vitro*. Cytotechnology.

[154] Lee S.H., Yeon Y.J. (2015). Efficacy and safety of a dieckol-rich extract (AG-dieckol) of brown algae, *Ecklonia cava*, in a pre-diabetic individuals: A double-blind, randomized, placebo-controlled clinical trial. Food Funct..

[155] Celikler S., Tas S., Vatan O., Ziyanok-Ayvalik S., Yildiz G., Bilaloglu R. (2009). Anti-hyperglycemic and anti-genotoxic potential of *Ulva rigida* ethnolic extract in the experimental diabetes mellitus. Food Chem. Toxicol..

[156] Motshakeri M., Ebrahimi M., Goh Y.M., Matanjun P., Mohamed S. (2013). *Sargassum polycystum* reduces hyperglycaemia, dyslipidaemia and oxidative stress via increasing insulin sensitivity in a rat model of type 2 diabetes. J. Sci. Food Agric..

[157] Tonks N.K. (2003). PTP1B: From the sidelines to the front lines!. FEBS Lett..

[158] Lee S., Wang Q. (2007). Recent development of small molecular specific inhibitor of protein tyrosine phosphatase 1B. Med. Res. Rev..

[159] Shi D., Xu F., He J., Li J., Fan X., Han J. (2008). Inhibition of bromophenols against PTP1B and anti-hyperglycemic effect of *Rhodomela confervoides* extract in diabetic rats. Chin. Sci. Bull..

[160] Liu X., Li X., Gao L., Cui C., Li C., Li J., Wang B. (2011). Extraction and PTP1B inhibitory activity of bromophenols from the marine red alga *Symphyocladia latiuscula*. Chin. J. Oceanol. Limnol..

[161] Qin J., Su H., Zhang Y., Gao J., Zhu L., Wu X., Pan H., Li X. (2010). Highly brominated metabolites from marine red alga *Laurencia similis* inhibit protein tyrosine phosphatase 1B. Bioorganic Med. Chem. Lett..

[162] Son Y.K., Jin S.E., Kim H.R., Woo H.C., Jung H.A., Choi J.S. (2011). Inhibitory activities of the edible brown alga *Laminaria japonica* on glucose-mediated protein damage and rat lens aldose reductase. Fish. Sci..

[163] Jung H.A., Yoon N.Y., Woo M.H., Choi J.S. (2008). Inhibitory activities of extracts from several kinds of seaweeds and phlorotannins from the brown alga *Ecklonia stolonifera* on glucose-mediated protein damage and rat lens aldose reductase. Fish. Sci..

[164] Islam M.N., Choi S.H., Moon H.E., Park H.A., Woo H.C., Choi J.S. (2014). The inhibitory activities of the edible green alga *Capsosiphon fulvescens* on rat lens aldose reductase and advanced and advanced glycation end products formation. Eur. J. Nutr..

[165] Kang C., Yin J.B., Lee H., Cha M., Sohn E.T., Moon J., Park C., Chun S., Jung E.S., Hing J.S. (2010). Brown alga *Ecklonia cava* attenuates type 1 diabetes by activating AMPK and Akt signaling pathways. Food Chem. Toxicol..

[166] Lee S.H., Kang S.M., Ko S.C., Lee D.H., Jeon Y.J. (2012). Octaphlorethol A, a novel phenolic compound isolated from a brown alga, *Ishige foliacea*, increases glucose transport 4-mediated glucose uptake in skeletal muscle cells. Biochem. Biophys. Res. Commun..

[167] Maeda H., Hosokawa M., Sashima T., Murakami-Funayama K., Miyashita K. (2009). Anti-obesity and anti-diabetic effects of fucoxanthin on diet-induced obesity conditions in a murine model. Mol. Med. Rep..

[168] Kang S.I., Jin Y.J., Ko H.C., Choi S.Y., Hwang J.H., Whang I., Kim M.H., Shin S.H., Jeong H.B., Kim S.J. (2008). Petalonia improves glucose homeostasis in streptozotocin-induced diabetic mice. Biochem. Biophys. Res. Commun..

[169] Kim M.J., Kim H.K. (2012). Insulinotrophic and hypolipidemic effects of *Ecklonia cava* in streptozotocin-induced diabetic mice. Asian Pac. J. Trop. Med..

[170] Lee S.H., Min K.H., Han J.S., Lee D.H., Park D.B., Jung W.K., Park P.J., Jeon B.T., Kim S.K., Jeon Y.J. (2012). Effects of brown alga, *Ecklonia cava* on glucose and lipid metabolism in C57BL/KsJ-db/db mice, a model of type 2 diabetes mellitus. Food Chem. Toxicol..

[171] Ramana K.V., Friedrich B., Srivastava S., Bhatnagar A., Srivastava S.K. (2004). Activation of nuclear factor-κB by hyperglycemia in vascular smooth muscle cells is regulated by aldose reductase. Diabetes.

[172] Obsorova I.G., Pacher P., Szabo C., Zsengeller Z., Hirooka H., Stevens M.J., Yorek M.A. (2005). Aldose reductase inhibition counteracts oxidative-nitrosative stress and poly(ADP-ribose) polymerase activation in tissue sites for diabetes complications. Diabetes.

[173] Vedantham S., Ananthakrishnan R., Schmidt A.M., Ramasamy R. (2012). Aldose reductase, oxidative stress and diabetic cardiovascular complications. Cardiovasc. Hematol. Agents Med. Chem..

[174] Tang J., Du Y., Petrash J.M., Sheibani N., Kern T.S. (2013). Deletion of Aldose Reductase from Mice Inhibits Diabetes-Induced Retinal Capillary Degeneration and Superoxide Generation. PLoS ONE.

[175] Tarantola E., Bertone V., Milanesi G., Capelli E., Ferrigno A., Neri D., Vairetti M., Barni S., Freitas I. (2012). Dipeptidylpeptidase-IV, a key enzyme for the degradation of incretins and neuropeptides: Activity and expression in the liver of lean and obese rats. Eur. J. Histochem..

[176] Mentlein R. (2009). Mechanisms underlying the rapid degradation and elimination of the incretin hormones GLP-1 and GIP. Best Pract. Res. Clin. Endocrinol. Metab..

[177] Garber A.J. (2011). Incretin effects on beta-cell function, replication and mass: The human perspective. Diabetes Care.

[178] Khan M.A., Deaton C., Rutter M.K., Neyses L., Mamas M.A. (2013). Incretins as a novel therapeutic strategy in patients with diabetes and heart failure. Heart Fail. Rev..

[179] Cormont M., Tanti J.F., Zahraoui A., van Obberghen E., Tavitian A., Marchand-Brustel Y.L. (1993). Insulin and okadaic acid induce Rab4 redistribution in adipocytes. J. Biol. Chem..

[180] Ozcan U., Yilmaz E., Ozcan L., Furuhashi M., Vailancourt E., Smith R.O., Gorgun C.Z., Hotamisligil G.S. (2006). Chemical chaperones reduce ER stress and restore glucose homeostasis in a mouse model of type 2 diabetes. Science.

[181] Sheetz M.J., King G.L. (2002). Molecular understanding of hyperglycemia’s adverse effects for diabetic complications. JAMA.

[182] Dugani C.B., Randhawa V.K., Cheng A.W., Patel N., Klip A. (2008). Selective regulation of the perinuclear distribution of glucose transport 4 (GLUT4) by insulin signals in muscle cells. Eur. J. Cell. Biol..

[183] Lin Y.C., Hung C.M., Tsai J.C., Lee J.C., Chen Y.L., Wei C.W., Kao J.Y., Way T.D. (2010). Hispidulin potently inhibits human glioblastoma multiforme cells through activation of AMP-activated protein kinase (AMPK). J. Agric. Food Chem..

[184] Hwang J.T., Kwon D.Y., Yoon S.H. (2009). AMP-activated protein kinase: A potential target for the diseases prevention by natural occurring polyphenols. New Biotechnol..

[185] Konrad D., Rudich A., Bilan P.J., Patel N., Richardson C., Witters L.A., Klip A. (2005). Troglitazone causes acute mitochondrial membrane depolarization in muscle cells. Diabetologia.

[186] Zhou M.H., Kirkpatrick S.S., Davis B.J., Nelson J.S., Wiles W.G., Schlattner U., Neumann D., Brownlee M., Freeman M.B., Goldman M.H. (2004). Activation of the AMP-activated protein kinase by the anti-diabetic drug metformin *in vivo* role of mitochondrial reactive nitrogen species. J. Biol. Chem..

[187] Tan M.H. (2000). Current treatment of insulin resistance in type 2 diabetes mellitus. Int. J. Clin. Pract. Suppl..

[188] Yuan Y.V., Walsh N.A. (2006). Antioxidant and antiproliferative activities of extracts from a variety of edible seaweeds. Food Chem. Toxicol..

[189] Balboa E.M., Conde E., Moure A., Falque E., Dominguez H. (2013). *In vitro* anti-oxidant properties of crude extracts and compounds from brown algae. Food Chem..

[190] Schinner S., Scherbaum W.A., Bornstein S.R., Barthel A. (2005). Molecular mechanisms of insulin resistance. Diabet. Med..

[191] Davi G., Falco A., Patrono C. (2005). Lipid peroxidation in diabetes mellitus. Antioxid. Redox Signal..

[192] Ray A., Huisman M.V., Tamsma J.T., van Asten J., Bingen B.O., Broeders E.A., Hoogeveen E.S., van Hout F., Kwee V.A., Research and Writing-Group (2009). The role of inflammation on artherosclerosis, intermediate and clinical cardiovascular endpoints in type 2 diabetes mellitus. Eur. J. Intern. Med..

[193] Chetyrkin S., Mathis M., Pedchenko V., Sanchez O.A., McDonald W.H., Hachey D.L., Madu H., Stec D., Hudson B., Voziyan P. (2011). Glucose autoxidation induces functional damage to proteins via modification of critical arginine residues. Biochemistry.

[194] Ceriello A. (2011). 25 years of progress in type 2 diabetes. Medicographia.

[195] Shoelson S.E., Lee J., Goldfine A.B. (2006). Inflammation and insulin resistance. J. Clin. Investig..

[196] King G.L. (2008). The role of inflammatory cytokines in diabetes and its complications. J. Periodontol..

[197] Lee S.H., Heo S.J., Hwang J.Y., Han J.S., Jeon Y.J. (2010). Protective effects of enzymatic digest from *Ecklonia cava* against high glucose-induced oxidative stress in human umbilical vein endothelial cells. J. Sci. Food. Agric..

[198] Lee S.H., Han J.S., Heo S.J., Hwang J.Y., Jeon Y.J. (2010). Protective effects of dieckol isolated from *Ecklonia cava* against high glucose-induced oxidative stress in human umbilical vein endothelial cells. Toxicol. Vitro.

[199] Gutteridge J.M. (1995). Lipid peroxidation and antioxidants as biomarkers of tissue damage. Clin. Chem..

[200] Bhatia S., Shukla R., Venkata Madhu S., Kaur Gambhir J., Madhava Prabhu K. (2003). Antioxidant status, lipid peroxidation and nitric oxide end products in patients of type 2 diabetes mellitus with nephropathy. Clin. Biochem..

[201] Kim K.B.W.R., Jung J.Y., Cho J.Y., Ahn D.H. (2012). Lipase inhibitory activity of ethyl acetate fraction from *Ecklonia cava* extracts. Biotechnol. Bioprocess Eng..

[202] Eom S.H., Lee M.S., Lee E.W., Kim Y.M., Kim T.H. (2013). Pancreatic lipase inhibitory activity of phlorotannins isolated from *Eisenia bicyclis*. Phytother. Res..

[203] Kim M.M., Rajapakse N., Kim S.K. (2009). Anti-inflammatory effect of *Ishige okamurae* ethanolic extract via inhibition of NF-kappaB transcription factor in RAW 264.7 cells. Phytother. Res..

[204] Lee S.H., Kang S.M., Ko S.C., Kang M.C., Jeon Y.J. (2013). Octaphlorethol A, a novel phenolic compound isolated from *Ishige foliacea*, protects against streptozotocin-induced pancreatic β-cell damage by reducing oxidative stress and apoptosis. Food Chem. Toxicol..

[205] Lee S.H., Park M.H., Park S.J., Kim J., Kim Y.T., Oh M.C., Jeong Y., Kim M., Han J.S., Jeon Y.J. (2012). Bioactive compounds extracted from Ecklonia cava by using enzymatic hydrolysis protects high glucose-induced damage in INS-1 pancreatic β-cells. Appl. Biochem. Biotechnol..

[206] Kang M.C., Wijesinghe W.A., Lee S.H., Kang S.M., Ko S.C., Yang X., Kang N., Jeon B.T., Kim J., Lee D.H. (2013). Dieckol isolated from brown seaweed *Ecklonia cava* attenuates type II diabetes in db/db mouse model. Food Chem. Toxicol..

[207] Maeda H., Hosokawa M., Sashima T., Funayama K., Miyashita K. (2005). Fucoxanthin from edible seaweed *Undaria pinnatifida*, shows anti-obesity effect through UCP1 expression in white adipose tissues. Biochem. Biophys. Res. Commun..

[208] Maeda H., Tsukui T., Sashima T., Hosokawa M., Miyashita K. (2008). Seaweed carotenoid, fucoxanthin, as a multi-functional nutrient. Asia Pac. J. Clin. Nutr..

[209] Hosokawa M., Miyashita T., Nishikawa S., Tsukui T., Beppu F., Okada T., Miyashita K. (2010). Fucoxanthin regulates adipocytokine mRNA expression in white adipose tissue of diabetic/obese KK-A^y^ mice. Arch. Biochem. Biophys..

[210] Kang S.I., Kim M.H., Shin H.S., Kim H.M., Hong Y.S., Park J.G., Ko H.C., Lee N.H., Chung W.S., Kim S.J. (2010). A water-soluble extract of *Petalonia binghamiae* inhibits the expression of adipogenic regulators in 3T3-L1 pre-adipocytes and reduces adiposity and weight gain in rats fed a high-fat diet. J. Nutr. Biochem..

[211] Kang S.I., Shin H.S., Kim H.M., Yoon S.A., Kang S.W., Kim J.H., Ko H.C., Kim S.J. (2012). *Petalonia binghamiae* extract and its constituent fucoxanthin ameliorate high-fat diet-induced obesity by activating AMP-activated protein kinase. J. Agric. Food Chem..

[212] World Health Organisation Fact Sheet Number 311: Obesity and Overweight. http://www.webcitation.org/6XUVS1R65.

[213] Resnick H.E., Valsania P., Halter J.B., Lin X. (2000). Relation of weight gain and weight loss on subsequent diabetes risk in overweight adults. J. Epidemiol. Community Health.

[214] Ross R. (1993). Atherosclerosis- an inflammatory disease. N. Engl. J. Med..

[215] Iacobellis G., Ribaudo M.C., Zappaterreno A., Iannucci C.V., Leonetti F. (2005). Prevalence of uncomplicated obesity in an Italian obese population. Obes. Res..

[216] Kopelmen P.G. (2000). Obesity as a medical problem. Nature.

[217] Dandona P., Aljada A., Bandyopadhay A. (2004). Inflammation: The link between insulin resistance, obesity and diabetes. Trends Immunol..

[218] Weisberg S.P., McCann D., Desai M., Rosenbaum M., Leibel R.L., Ferrante A.W. (2003). Obesity is associated with macrophage accumulation in adipose tissue. J. Clin. Investig..

[219] Xu H., Barnes G.T., Yang Q., Tan G., Yang D., Chou C.J., Sole J., Nichols A., Ross J.S., Tartaglia L.A. (2003). Chronic inflammation in fat plays a crucial role in the development of obesity-related insulin resistance. J. Clin. Investig..

[220] Sartipy P., Loskutoff D.J. (2003). Monocyte chemoattractant protein 1 in obesity and insulin resistance. Proc. Natl. Acad. Sci. USA.

[221] Ruan H., Hacohen N., Golub T.R., van Raijs L., Lodish H.F. (2002). Tumor necrosis factor-α suppresses adipocyte-specific genes and activates expression of pre-adipocyte genes in 3T3-L1 adipocytes: Nuclear factor-κB activation by TNF-α is obligatory. Diabetes.

[222] Hotamisligil G.S., Shargill N.S., Spiegelman B.M. (1993). Adipose expression of tumor necrosis factor-α: Direct role in obesity-linked insulin resistance. Science.

[223] Krogh-Madsen R., Plomgaard P., Moller K., Mittendorfer B., Pedersen B.K. (2006). Influence of TNFα and IL6 infusions on insulin sensitivity and expression of IL-18 in humans. Am. J. Physiol. Endocrinol. Metab..

[224] Cancello R., Henegar C., Viguerie N., Taleb S., Poitou C., Rouault C., Coupaye M., Pelloux V., Hugol D., Bouillot J.L. (2005). Reduction of macrophage infiltration and chemoattractant gene expression changes in white adipose tissue of morbidly obese subjects after surgery-induced weight loss. Diabetes.

[225] Otto T.C., Lane M.D. (2005). Adipose development: From stem cell to adipocyte. Crit. Rev. Biochem. Mol. Biol..

[226] Pickup J.C., Chusney G.D., Thomaz S.M., Burt D. (2000). Plasma interleukin-6, tumor necrosis factor-α and blood cytokine production in type 2 diabetes. Life Sci..

[227] Moller D.E. (2000). Potential role of TNF-α in the pathogenesis of insulin resistance and Type 2 diabetes. Trends Endocrinol. Metab..

[228] Exel E.V., Gussekloo J., Craen A.J.M., Frolich M., Wiel A.B., Westendorp R.G.J. (2002). Low production capacity of interleukin-10 associates with metabolic syndrome and Type 2 diabetes. Diabetes.

[229] Haemmerle G., Lass A., Zimmermann R., Gorkiewicz G., Meyer C., Rozman J., Heldmaier G., Maier R., Theussl C., Eders S. (2006). Defective lipolysis and altered energy metabolism in mice lacking adipose triglyceride lipase. Science.

[230] Hotamisligil G.S., Arner P., Caro J.F., Atkinson R.L., Spiegelman B.M. (1995). Increased adipose tissue expression of tumor necrosis factor-α in human obesity and insulin resistance. J. Clin. Investig..

[231] Guilherme A., Virbasius J.V., Puri V., Czech M.P. (2008). Adipocyte dysfunctions linking obesity to insulin resistance and type 2 diabetes. Nat. Rev. Mol. Cell Biol..

[232] Kim K.T., Rioux L.E., Turgeon S.L. (2014). α-Amylase and α-glucosidase inhibition is differentially modulated by fucoidan obtained from *Fucus vesiculosus* and *Ascophyllum nodosum*. Phytochemistry.

[233] Apostolidis E., Karayannakis P.D., Kwon Y.I., Lee C.M., Seeram N.P. (2011). Seasonal variation of phenolic antioxidant-mediated α-glucosidase inhibition of *Ascophyllum nodosum*. Plant Foods Hum. Nutr..

[234] Gosch B.J., Paul N.A., de Nys R., Magnusson M. (2015). Seasonal and within-plant variation in fatty acid content and composition in the brown seaweed *Spatoglossum macrodontum* (Dictyotales, Phaeophyceae). J. Appl. Phycol..

